# DGKα/ζ inhibition lowers the TCR affinity threshold and potentiates antitumor immunity

**DOI:** 10.1126/sciadv.adk1853

**Published:** 2023-11-24

**Authors:** Rakeeb Kureshi, Elisa Bello, Courtney T.S. Kureshi, Michael J. Walsh, Victoria Lippert, Megan T. Hoffman, Michael Dougan, Tyler Longmire, Michael Wichroski, Stephanie K. Dougan

**Affiliations:** ^1^ Department of Immunology, Harvard Medical School, Boston, MA, USA.; ^2^ Department of Cancer Immunology and Virology, Dana-Farber Cancer Institute, Boston, MA, USA.; ^3^ Department of Gastroenterology, Massachusetts General Hospital, Boston, MA, USA.; ^4^ Bristol Myers Squibb, Cambridge, MA, USA.

## Abstract

Diacylglycerol kinases (DGKs) attenuate diacylglycerol (DAG) signaling by converting DAG to phosphatidic acid, thereby suppressing pathways downstream of T cell receptor signaling. Using a dual DGKα/ζ inhibitor (DGKi), tumor-specific CD8 T cells with different affinities (TRP1^high^ and TRP1^low^), and altered peptide ligands, we demonstrate that inhibition of DGKα/ζ can lower the signaling threshold for T cell priming. TRP1^high^ and TRP1^low^ CD8 T cells produced more effector cytokines in the presence of cognate antigen and DGKi. Effector TRP1^high^- and TRP1^low^-mediated cytolysis of tumor cells with low antigen load required antigen recognition, was mediated by interferon-γ, and augmented by DGKi. Adoptive T cell transfer into mice bearing pancreatic or melanoma tumors synergized with single-agent DGKi or DGKi and antiprogrammed cell death protein 1 (PD-1), with increased expansion of low-affinity T cells and increased cytokine production observed in tumors of treated mice. Collectively, our findings highlight DGKα/ζ as therapeutic targets for augmenting tumor-specific CD8 T cell function.

## Teaser

DGKα/ζ inhibition enhances activation and effector function of tumor-specific CD8 T cells.

## INTRODUCTION

CD8 T cells can induce direct cytolysis of cancer cells in an antigen-dependent manner (*1*, *2*). Across multiple tumor types, T cell density and T cell receptor (TCR) diversity are prognostic for overall survival (*3*). However, in many cases, CD8 T cells are sparsely found in tumors (*4*) or are dysfunctional with progressive loss of effector proteins interferon-γ (IFN-γ), tumor necrosis factor–α (TNFα), perforin, and granzyme B (*5*, *6*). Immune checkpoint blockade therapies enhance T cell function and improve antitumor immunity by blocking inhibitory receptors such as programmed cell death protein 1 (PD-1) (*7*–*10*). Yet, many patients with a range of tumor types are unresponsive. Response to checkpoint blockade is correlated with the presence of nonself-antigens, including neoantigens arising from tumor mutations and viral proteins expressed in tumors of viral origin (*11*). Many cancers have a low mutational burden; because T cells that recognize self-proteins with high affinity are deleted in the thymus, eliciting tumor-reactive T cells against tumors with few viral or neoantigens remains challenging (*12*). Therapies that enhance T cell priming to self-antigens overexpressed in tumors are needed to expand TCR diversity and provide immunotherapy options to a broader range of patients.

CD8 T cells recognize tumor antigens through engagement of their TCR with peptides loaded on major histocompatibility complex I (pMHC), and the affinity of this interaction affects downstream activation and effector function (*13*, *14*). Short-lived TCR:pMHC interactions do not stimulate sufficient activation of T cells (*15*, *16*). Alongside affinity, the avidity or number of TCR:pMHC molecular interactions engaged is instrumental in forming an immunological synapse (*17*, *18*). On average, TCRs recognizing tumor-associated self-antigens have a lower affinity for their antigens than TCRs recognizing viral antigens (*19*). Lower-affinity TCR interactions decrease activation and T cell cytolysis of tumor cells (*20*, *21*), whereas mutations that enhance TCR affinity for cognate antigen lead to greater cytokine production and cytolytic activity (*22*, *23*). Down-regulation of surface MHC-I expression on tumor cells (*24*) diminishes avidity, and reduced antigen presentation is a major cause of both primary and acquired resistance to immunotherapy (*25*, *26*).

TCR engagement with pMHC leads to phosphorylation of the CD3 complex and activation of phospholipase-Cγ that hydrolyzes phosphatidylinositol 4,5-bisphosphate to generate diacylglycerol (DAG) and inositol trisphosphate (IP_3_), which propagates downstream signaling (*27*). IP_3_ induces calcium flux into the cytosol and ultimately activation of the transcription factor nuclear factor of activated T cells (NFAT). DAG recruits Ras guanyl release protein 1 (RasGRP1) and protein kinase Cθ (PKCθ) (*28*, *29*) and propagates signaling that results in activation of activator protein 1 (AP-1) and canonical nuclear factor κB (ΝF-κB) pathways, respectively. Loss of DAG in T cells attenuates activation of AP-1 and NF-κB, essential transcription factors for interleukin-2 (IL-2), IFN-γ, TNFα, and granzyme B expression (*30*, *31*). Moreover, NFAT transcriptional activity in the absence of AP-1 induces anergy, highlighting the importance of DAG in regulation of T cell fate decisions (*32*).

DAG levels are negatively regulated through phosphorylation by a family of diacylglycerol kinases (DGK) that convert DAG to phosphatidic acid (*33*). DGKα and DGKζ are two isoforms most abundantly expressed in T cells that diminish downstream TCR signaling (*34*, *35*). Several studies have characterized the molecular and cellular roles of DGKα/ζ in T cells (*35*–*39*). Deficiency in either isoform yields greater downstream TCR signaling as assessed via enhancement in phosphorylation of extracellular signal–regulated kinase 1/2 (ERK1/2), whereas overexpression of DGKα attenuates downstream TCR signaling (*40*). Anergic or hyporesponsive T cells have higher expression of DGKα (*40*, *41*). Kras overexpression bypasses upstream DGKα and rescues proliferative and cytokine capacity in anergic T cells, suggesting that DGKα maintains T cell anergy (*41*). In the context of viral infection, lymphocytic choriomeningitis virus–specific T cells lacking either isoform of DGK exhibited greater cytokine production 7 days postinfection and ultimately cleared viral infection better than wild-type (WT) T cells (*42*). In a short hairpin RNA–based screen for genes that, when silenced, increase T cell accumulation in tumors, genes encoding both DGKα and DGKζ scored in the top list of candidates (*43*). Moreover, DGKζ-deficient mice exhibited greater control of MC38 tumors (*44*). DGKα is up-regulated in hypofunctional OT-I T cells in B16 ovalbumin (OVA)-expressing tumors and therapeutic inhibition of DGKα combined with PD-1 blockade for improved tumor control (*45*). However, current DGK inhibitors are suboptimal therapeutics as they target the α but not ζ isoform, which may have compensatory functions. In addition, current DGKα inhibitors have low affinity (median inhibitory concentration > 1 μM) and have off-target binding to the serotonin receptor (*46*). These data demonstrate the potential for DGKα/ζ inhibition to improve tumor-specific CD8 T cell function. Yet, how DGKα/ζ inhibition can modulate tumor-specific T cell activity in the context of varying TCR affinities or limited antigen presentation remains underexplored.

Here, we use a potent DGKα/ζ lipid kinase inhibitor to enhance tumor-specific CD8 T cell activation and effector functions (*47*). We use TRP1^high^ and TRP1^low^ transnuclear mice bearing CD8 T cells that recognize an epitope from the melanoma self-antigen tyrosinase-related protein 1 (Tyrp1) with different affinities (*48*). We demonstrate that DGKi enhances priming and antigen-specific effector functions of both T cell clones in vitro and in vivo that is most pronounced in situations where TCR affinity or avidity are suboptimal.

## RESULTS

### DGKi augments priming of TRP1^high^ and TRP1^low^ CD8 T cells

**Fig. 1.**
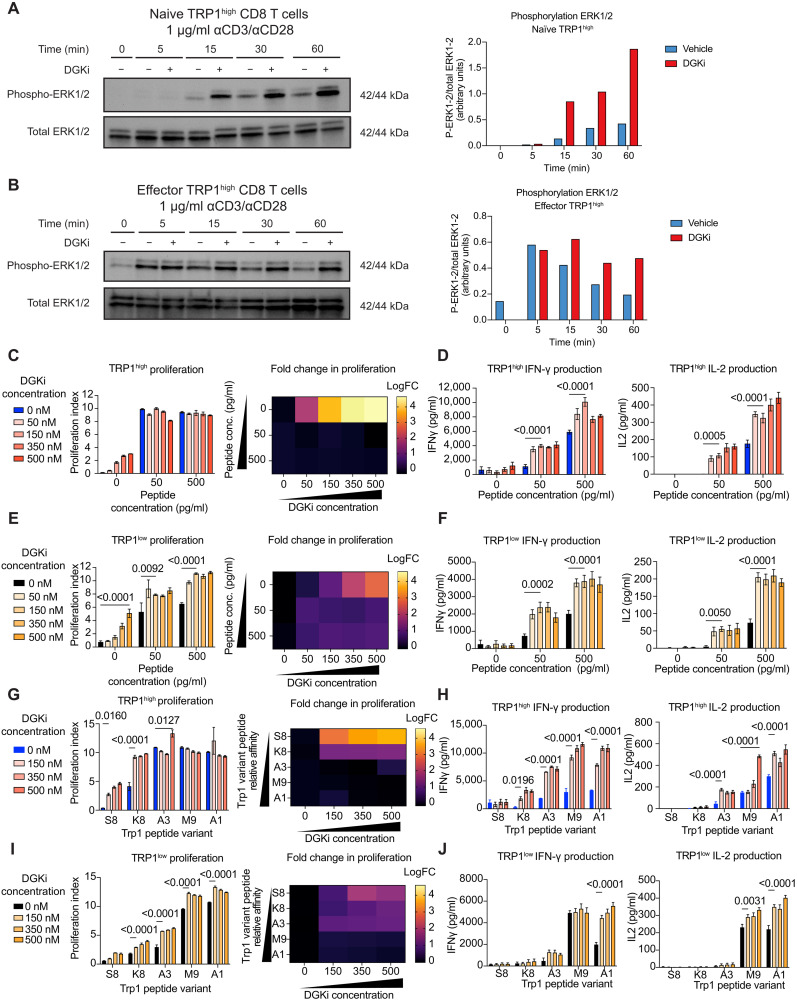
Inhibition of DGKα/ζ in antigen-specific CD8 T cells enhances proliferation and cytokine production in a dose-dependent manner. (**A**) Unactivated or (**B**) effector TRP1^high^ CD8 T cells were activated for 60 min on αCD3/28 (1 μg/ml)–coated plates for 5, 15, 30, and 60 min followed by cell lysis and ultimately immunoblot analysis. Total ERK1/2 served as a loading control. Band intensities were quantified with ImageJ. (**C**) CTV-labeled TRP1^high^ or (**E**) TRP1^low^ CD8 T cells were cocultured with B cells treated with native Trp1 peptide at the indicated concentration with and without varying concentrations of the DGK inhibitor, and proliferation indices were assessed via flow (*n* = 4). Log fold change was calculated in reference to the vehicle-treated group for each peptide concentration. Supernatants were collected after 72 hours, and IFN-γ and IL-2 production was measured via ELISA for (**D**) TRP1^high^ or (**F**) TRP1^low^ (*n* = 4). (**G**) TRP1^high^ or (**I**) TRP1^low^ CD8 T cells were cocultured with B cells treated with A1, A3, M9, K8, or S8 Trp1 peptide variants at 50 pg/ml (±DGK inhibitor) for 84 hours (*n* = 4). Proliferation indices were quantified via flow cytometry. Log fold change was calculated in reference to vehicle-treated group for each respective peptide variant. Supernatants were collected after 84 hours, and IFN-γ and IL-2 production was assessed via ELISA for (**H**) TRP1^high^ or (**J**) TRP1^low^ cells (*n* = 4). Error bars represent SEM. Proliferation indices and cytokine production was compared with a two-way ANOVA and Sidak’s test for multiple comparisons. *P* values are denoted above groups being compared.

To address the effect of inhibiting DGKα/ζ in tumor-specific T cells, we used TRP1^high^ and TRP1^low^ transnuclear mice, originally generated by somatic cell nuclear transfer from two different CD8 T cells recognizing TRP1 peptide presented by H2-D^b^ (*48*). Although these two TCR clones recognize the same peptide-MHC complex, they do so with different affinities, as previously reported (*14*, *48*, *49*). We first confirmed that DGKi affects TCR signaling in both naïve CD8 T cells and effector CD8 T cells. Effector TRP1^high^ CD8 T cells were differentiated over the course of 8 days in vitro. Naïve or effector TRP1^high^ CD8 T cells were activated with αCD3/28 in the presence or absence of 100 nM DGKi followed by analysis of phosphorylated Erk1/2 by immunoblot. We observed an enhancement in phosphorylated Erk1/2 upon inhibition of DGKα/ζ in naïve TRP1^high^ CD8 T cells, and we also saw sustained phosphorylated Erk1/2 in effector TRP1^high^ CD8 T cells (Fig. 1, A and B). This demonstrates augmentation of the mitogen-activated protein kinase (MAPK) pathway downstream of DAG upon treatment with DGKi. Other pathways activated downstream of TCR stimulation, such as mammalian target of rapamycin complex 1/2 (mTORC1/2), were largely unaffected by DGKi treatment (fig. S1).

We next assessed whether CD8 T cells exhibit more robust priming in the presence of DGKi. We quantified T cell priming by measuring proliferation and production of the cytokines IL-2 and IFN-γ. TRP1^high^ or TRP1^low^ CD8 T cells were labeled with CellTraceViolet (CTV) and cocultured with antigen-presenting cells pulsed with titrated concentrations of the cognate Trp1 peptide and DGKi. Higher-affinity TRP1^high^ CD8 T cells primed with Trp1 peptide proliferated maximally with or without DGKi; however, we observed an enhancement in basal proliferation with IL-2–supplemented media and DGKi that was not seen when T cells had neither peptide nor IL-2 present (Fig. 1C and figs. S2 and S3). TRP1^high^ T cells activated in the presence of DGKi and cognate peptide produced greater amounts of IL-2 and IFN-γ, with no production of either cytokine in the absence of peptide (Fig. 1D). Moreover, we observed temporal enhancement of cytokines at both the transcriptional and protein level in the presence of DGKi (fig. S4). Lower-affinity TRP1^low^ T cells showed an increased fraction of proliferating cells in the presence of DGKi with a greater augmentation by DGKi at lower concentrations of peptide (Fig. 1E and fig. S2). TRP1^low^ T cells also produced greater quantities of IL-2 and IFN-γ in the presence of DGKi and cognate peptide (Fig. 1F). As observed previously (*23*, *50*), induction of IFN-γ production by both T cell clonotypes required less peptide simulation than did IL-2. Together, these data demonstrate that inhibition of DGKα/ζ activity at the time of priming enhances T cell proliferation and cytokine production. Moreover, DGKi had a more profound effect on proliferation of lower-affinity TCR clones, demonstrating the potential impact of DGKi in overcoming a threshold required for activation of low-affinity tumor-specific T cells.

Myc levels are digitally tuned by TCR signaling and impact proliferation (*51*). TRP1^high^ mice were crossed with Myc–green fluorescent protein (GFP) mice, and CTV-labeled TRP1^high^;Myc-GFP CD8 T cells were isolated and stimulated in vitro with αCD3/28 for 24, 48, and 72 hours. DGKi increased both the frequency of Myc^+^ cells and the per-cell level of Myc protein in CD8 T cells at early time points (fig. S5, B and C). Consistent with previous studies (*52*), we observed a temporal control of Myc expression independent of cell division in both vehicle and DGKi (fig. S5, D and E). These data suggest that DGKi augments the initial burst in Myc protein levels and contributes to increased proliferation.

To test the effects of DGKi across a wider range of TCR affinities, we used a previously reported series of altered peptide ligands (A1, M9, A3, K8, and S8) that have been shown to elicit variable activation of both Trp1 CD8 T cell clones, with S8 inducing no activation (*14*, *49*). TRP1^high^ T cells vigorously proliferate in the presence of agonist peptides A3, M9, and A1, with no further augmentation by DGKi. On the other hand, the fraction of TRP1^high^ cells that proliferated in response to the lower-affinity peptide K8 and the null peptide S8 was greatly enhanced by DGKi. Fold change analysis shows that DGKi can better augment proliferation against lower-affinity peptide variants (Fig. 1G). IFN-γ production was enhanced by DGKi for all peptides except S8 (Fig. 1H). IL-2 production increased with DGKi in the presence of A3, M9, or A1 peptide, consistent with IL-2 having a more stringent requirement for higher TCR affinity than IFN-γ (Fig. 1H). DGKi increased proliferation of TRP1^low^ T cells across the entire peptide series (Fig. 1I). Similar to our findings with TRP1^high^ T cells, fold change analysis demonstrates a greater DGKi-mediated enhancement of TRP1^low^ proliferation against lower-affinity peptides (Fig. 1I). DGKi also increased expression of IFN-γ and IL-2 from TRP1^low^ cells cultured with M9 or A1 peptides (Fig. 1J). Collectively, these data suggest that DGKi reduces the affinity threshold for TRP1^high^ and TRP1^low^ priming. DGKi-mediated increases in proliferation require the least amount of stimulus, whereas enhancement of IL-2 production requires increasing peptide concentrations or affinity.

### DGKi enhances T cell–mediated killing of tumor cells when antigen is limited

**Fig. 2.**
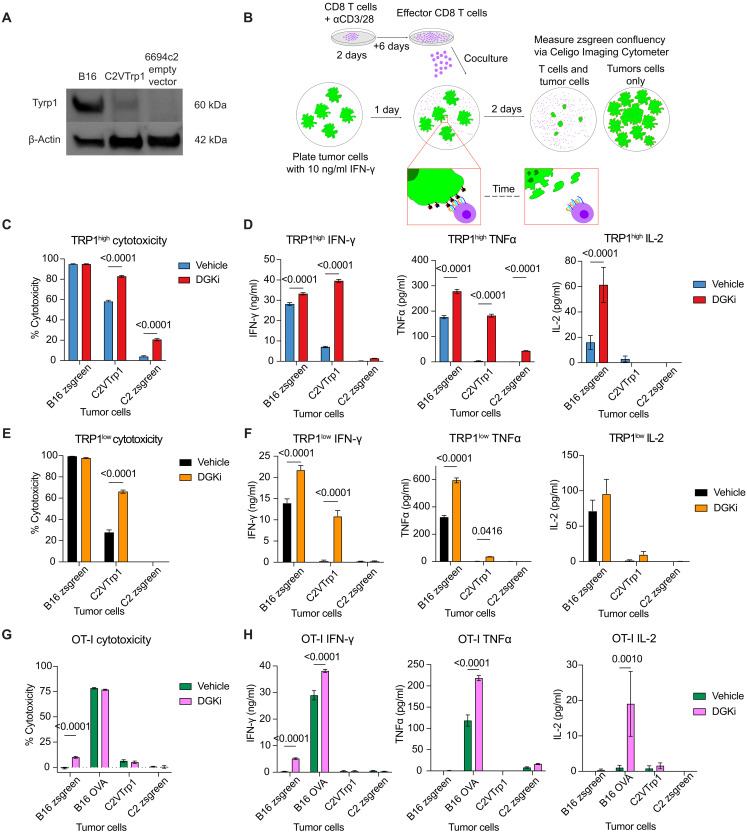
DGKα/ζ inhibition in CD8 T cells enhances killing of pancreatic cancer cells. (**A**) Immunoblot analysis of total protein lysates collected from B16, C2VTrp1, and 6694c2 cells. Total cellular lysates were blotted for Tyrp1 protein to determine protein expression level with β-actin serving as a loading control. (**B**) Schematic of cytotoxicity assay. Effector CD8 T cells were differentiated in vitro for 8 days followed by coculture with tumor cells pretreated with IFN-γ. Pretreated IFN-γ media were removed from tumors before coculture in fresh media containing CD8 T cells. (**C**) TRP1^high^, (**E**) TRP1^low^, or (**G**) OT-I effector CD8 T cells were cocultured with indicated zsgreen-expressing tumor cells at a 5:1 (effector:tumor) ratio for 48 hours with or without 100 nM DGKi (*n* = 8). zsgreen confluency was measured with a Celigo Imaging Cytometer, and % cytotoxicity was quantified by the change in confluency compared to tumor-only control wells. Error bars represent SEM. Supernatants were collected after 48 hours and IL-2, IFN-γ, and TNFα production was measured via ELISA for (**D**) TRP1^high^, (**F**) TRP1^low^, or (**H**) OT-I T cell groups (*n* = 8). Error bars represent SEM. Cytotoxicity and cytokine production were compared with a two-way ANOVA with Sidak’s test for multiple comparisons. Statistically significant *P* values are denoted above compared groups.

Melanoma endogenously expresses Tyrp1 protein and can be killed in vitro by TRP1^high^ and TRP1^low^ effector CD8 T cells (*53*). Effector cell recognition of tumor pMHC again requires signaling through the TCR. To determine whether DGKi enhances suboptimal TCR signaling in effector CD8 T cells, we used both B16 melanoma cells and the pancreatic cancer cell line 6694c2 transduced to express low amounts of Tyrp1 (C2VTrp1) (Fig. 2A). Although we cannot measure Trp1 pMHC complexes directly, we hypothesize that lower total amounts of Trp1 protein in C2VTrp1 cells results in decreased pMHC density and decreased avidity of TCR pMHC interactions.

To assess T cell–directed killing of tumor cells, we developed an in vitro T cell cytotoxicity assay using zsGreen-expressing tumor cells (Fig. 2B). TRP1^high^ effector T cells were cocultured with B16, C2VTrp1, or 6694c2 cells. We observed maximal killing of B16 cells, which could not be further augmented by DGKi (Fig. 2C). TRP1^high^ cells treated with vehicle modestly killed C2VTrp1 and had no effect on parental 6694c2 tumor cells. Addition of DGKi markedly increased cytotoxicity of C2VTrp1 and 6694c2 parental tumor cells, indicating that DGKi enhances cytotoxicity of effector CD8 T cells in conditions where antigen is limited (Fig. 2C). DGKi did not affect viability of B16 or C2VTrp1 cells in the absence of T cells and DGKi-mediated killing of C2VTrp1 saturated at DGKi concentrations above 100 nM (fig. S6).

Effector CD8 T cell production of cytokines IFN-γ and TNFα is correlated with antitumor immunity. We observed a more pronounced DGKi-mediated increase in production of IFN-γ and TNFα by TRP1^high^ T cells when cocultured with C2VTrp1 and a modest enhancement of cytokine production when cultured with B16 cells (Fig. 2D). IL-2 production was also increased by DGKi when TRP1^high^ T cells were cocultured with B16 cells (Fig. 2D), consistent with previous findings that a greater concentration of peptide is required to induce IL-2 production than is required for IFN-γ and TNFα (*23*, *49*). DGKi increased IL2, IFN-γ, and TNFα levels at both the transcription and protein level after 24 hours of coculture of TRP1^high^ and C2VTrp1 tumor cells (fig. S7, A to C). On a per-cell basis, effector CD8 T cells stimulated with αCD3/CD28 produced more IFN-γ and IL-2 in the presence of DGKi as determined by intracellular cytokine staining (fig. S7D).

Similar to its effects on TRP1^high^ cells, DGKi enhanced TRP1^low^ cytotoxicity against C2VTrp1 cells but not against B16 tumor cells with higher antigen density (Fig. 2E). TRP1^low^ IFN-γ and TNFα production in response to B16 and C2VTrp1 were enhanced by DGKi (Fig. 2F). To benchmark these results against a commonly used high-affinity TCR, we used OT-I T cells that recognize an epitope from the model antigen OVA. Upon coculture of OT-I T cells with B16 OVA, B16, C2VTrp1, or 6694c2 cells, we observed robust killing of B16 OVA cells with or without DGKi. Although OT-I T cells did not kill parental B16 tumor cells, we observed a small enhancement in cytotoxicity with DGKi (Fig. 2G). DGK inhibition increased OT-I production of IFN-γ, TNFα, and IL-2, when cocultured with B16 OVA cells (Fig. 2H). Together, these results demonstrate that DGKi enhances cytotoxicity in conditions where antigen is limited but increased cytokine production from both low- and high-avidity interactions.

### DGKi-mediated cytotoxicity is dependent on MHC-I and IFN-γ expression

**Fig. 3.**
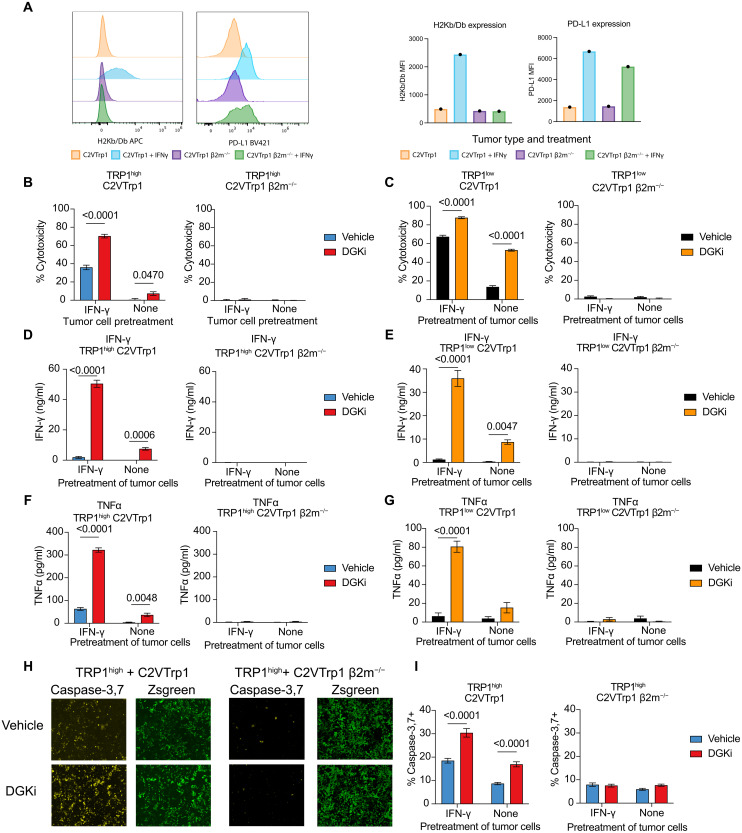
DGKi-mediated cytotoxicity by CD8 T cells occurs even at very low peptide MHC densities. (**A**) C2VTrp1 were transfected with a px459 Cas-9–expressing vector and a single guide RNA–targeting β2m. MHC-I and PD-L1 expression was assessed in untreated and IFN-γ–treated (24 hours) C2VTrp1 WT and β2m^−/−^ via flow cytometry staining of H2K^b^/D^b^ and PD-L1 with corresponding mean fluorescence intensity (MFI) values quantified. C2VTrp1 WT and β2m^−/−^ lines were cultured for 24 hours ± IFN-γ (10 ng/ml) before coculture with effector (**B**) TRP1^high^ or (**C**) TRP1^low^ CD8 T cells. TRP1^high^ and TRP1^low^ experiments were performed independently. T cells and tumor cells were cocultured for 48 hours before imaging on a Celigo Imaging Cytometer (*n* = 8). Supernatants were collected after 48 hours of coculture and (**D** and **E**) IFN-γ and (**F** and **G**) TNFα protein levels were quantified via ELISA (*n* = 8). TRP1^high^ CD8 T cells were cocultured with C2VTrp1 WT or β2m^−/−^ tumor cells in the presence of a caspase-3 reporter dye for 48 hours. (**H**) Representative well images of caspase-3 activity (yellow) and zsGreen (green) expression after 48 hours of coculture between TRP1^high^ T cells and indicated tumor cells. (I) Quantification of data shown in H (*n* = 8). Error bars represent SEM. Caspase-3^+^ activity was compared with a two-way ANOVA with Sidâk’s test for multiple comparison. Statistically significant *P* values are denoted above compared groups.

We generated MHC-I–deficient C2VTrp1 cells by CRISPR-Cas9 deletion of *B2m*. Similar to their parental 6694c2 cells (*54*), C2VTrp1 cells express basal levels of surface MHC-I and programmed death ligand 1 (PD-L1) but rapidly up-regulate expression of both upon treatment with IFN-γ. No surface MHC-I expression was observed in the presence of IFN-γ in C2VTrp1 cells deficient for β2m but up-regulation of PD-L1 upon IFN-γ treatment remained (Fig. 3A). We cocultured TRP1^high^ or TRP1^low^ T cells with C2VTrp1 with varying levels of MHC-I expression: high MHC-I expression (IFN-γ pretreatment), basal MHC-I expression (no IFN-γ pretreatment), or no MHC-I expression (β2m^−/−^). DGKi treatment augmented cytotoxicity of TRP1^high^ and TRP1^low^ T cells against C2VTrp1 cells with or without IFN-γ, but no cytotoxicity was observed against C2VTrp1;β2m^−/−^ cells under any condition (Fig. 3, B and C). Evaluation of IFN-γ and TNFα cytokine production from these cultures revealed similar DGKi-mediated augmentation of cytokine production against C2VTrp1 cells with or without IFN-γ pretreatment, but no cytokines were observed in response to C2VTrp1;β2m^−/−^ cells (Fig 3, D to G). These results confirm that although DGKi decreases the threshold for TCR activation, activation is still dependent on pMHC.

CD8 T cells can induce caspase-mediated cell death that converges on caspase-3 (*55*). Using a caspase-3 reporter dye, we found that DGKi increases the fraction of caspase-3^+^ tumor cells after coculture with TRP1^high^ T cells when tumor cells express MHC-I. We observed only background levels of caspase-3^+^ cells when TRP1^high^ T cells were cocultured with C2VTrp1 β2m^−/−^ cells, which was not augmented by DGKi (Fig. 3, H and I).

**Fig. 4.**
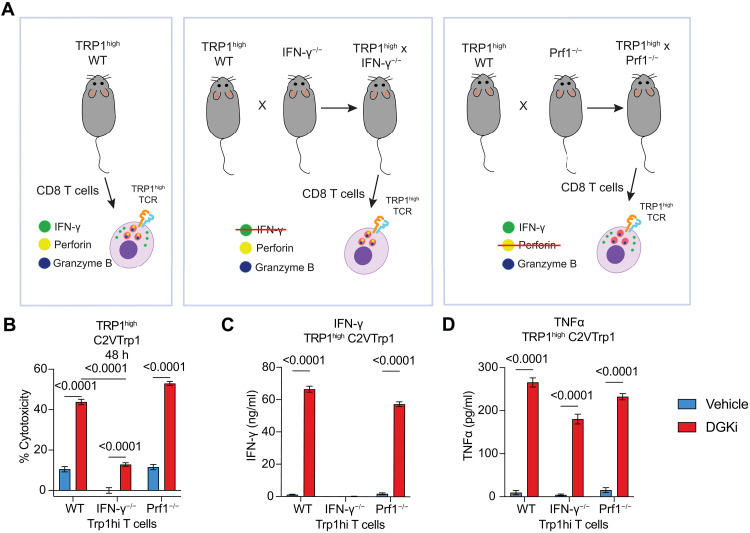
DGKi-dependent cytotoxicity is mediated by IFN-γ but not perforin. (**A**) TRP1^high^ transnuclear mice were crossed with IFN-γ^−/−^ or Prf1^−/−^ mice to generate mice bearing TRP1^high^ CD8 T cells deficient in either IFN-γ or Prf1. (**B**) C2VTrp1 cells were cocultured with wild-type (WT) TRP1^high^, IFN-γ^−/−^ TRP1^high^, or Prf1^−/−^ TRP1^high^ effector CD8 T cells for 48 hours before measuring cytotoxicity via the Celigo (*n* = 8). Supernatants were collected after 48 hours of coculture and (**C**) IFN-γ and (**D**) TNFα protein levels were measured via ELISA (*n* = 8). Error bars represent SEM. Statistical comparisons were conducted via a two-way ANOVA analysis with Sidâk’s test for multiple comparisons. Statistically significant *P* values are denoted above the groups compared. Findings are representative of two independent experiments.

CD8 T cell cytotoxicity can be mediated by perforin and granzyme B (*56*), but IFN-γ is also capable of inducing tumor cell cytostasis and death particularly in combination with TNFα. Given that DGKi increases IFN-γ and TNFα production, we aimed to determine the mechanism of cell death induced by Trp1-specific T cells in vitro. We crossed TRP1^high^ mice with either IFN-γ^−/−^ or Prf1^−/−^ mice to generate mice containing TRP1^high^ CD8 T cells deficient in IFN-γ or perforin (Fig. 4A). Upon coculture of WT, IFN-γ^−/−^, or Prf1^−/−^ TRP1^high^ CD8 T cells with C2VTrp1 tumor cells, we observed a decrease in cytotoxicity from TRP1^high^ T cells deficient in IFΝ-γ and no loss in cytotoxicity from TRP1^high^ T cells deficient in perforin (Fig. 4B). Notably, C2VTrp1 tumor cells were pulsed with IFN-γ before coculture to induce MHC-I expression, making differences in MHC-I an unlikely explanation for the reduced cytotoxicity from Trp1;IFN-γ^−/−^ T cells. Although DGKi enhanced cytotoxicity from all T cell genotypes, Trp1;IFN-γ^−/−^ cytotoxicity could not be fully rescued, indicating that IFN-γ is required for tumor cell killing in both vehicle and DGKi conditions. Consistent with prior observations, DGKi increased IFN-γ production from WT and TRP1^high^;Prf1^−/−^ T cells (Fig. 4C). TRP1^high^;IFN-γ^−/−^ T cells remain otherwise functional as noted by a small enhancement in cytotoxicity as well as robust production of TNFα production in the presence of DGKi (Fig. 4D).

### Early exposure to DGKi modestly imprints subsequent cell fate but is not required for DGKi enhancement of effector functions

**Fig. 5.**
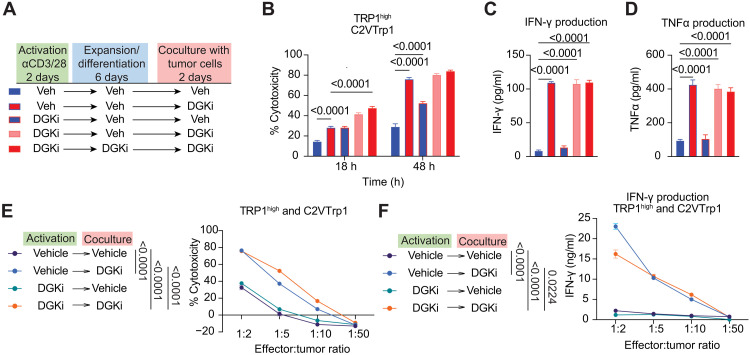
Timing of DGKi treatment affects TRP1^high^ cytotoxicity of C2VTrp1 cells. (**A**) TRP1^high^ were exposed to 100 nM DGKi at three phases: T cell activation (2 days), expansion/effector differentiation (6 days), and coculture with C2VTrp1 tumor cells (2 days). (**B**) TRP1^high^ CD8 T cells were cocultured with C2VTrp1 tumors, and cytotoxicity was determined at 18 and 48 hours. (**C**) IFN-γ and (**D**) TNFα production was measured via ELISA from supernatants after 48 hours of coculture with C2VTrp1 tumor cells. Error bars represent SEM. Cytotoxicity and cytokine production were compared with a two-way ANOVA and Sidak’s test for multiple comparisons. (**E**) TRP1^high^ CD8 T cells were activated in the presence or absence of 100 nM DGKi during activation and/or coculture with tumor cells. TRP1^high^ T cells were cocultured with C2VTrp1 tumors cells at indicated T cell:tumor cell ratios (*n* = 6). (**F**) IFN-γ production was measured from supernatants collected 48 hours after coculture. Area under the curve values representing total cytotoxicity and total IFN-γ production for each group were compared via one-way ANOVA and Tukey’s test for multiple comparisons. Statistically significant *P* values are denoted between compared groups.

CD8 T cell fate decisions can be imprinted during initial priming (*14*, *57*). We therefore asked whether exposure to DGKi during initial priming is sufficient to yield more robust effector T cells. TRP1^high^ CD8 T cells were treated with DGKi during three distinct time windows: activation with αCD3/CD28, expansion/differentiation, or during coculture with tumor cells (Fig. 5A). DGKi is a noncovalent inhibitor, which allows for restoration of DGK activity upon removal of the drug (*47*). Exposure to DGKi at the time of priming significantly increased the downstream cytolytic capacity of effector cells compared to T cells that had never seen DGKi, indicating that early exposure to DGKi can influence subsequent cell fate (Fig. 5B). However, exposure to DGKi during coculture with tumor cells significantly enhanced cytolysis and production of cytokines IFN-γ and TNFα, with little to no difference between cells that had seen DGKi at all three stages versus those that were exposed to DGKi only during the coculture window (Fig. 5, B to D). When we looked across a range of effector-to-target ratios, the presence of DGKi during coculture could elicit cytotoxicity and IFN-γ production even when tumor cells outnumbered T cells by 5:1 or 10:1, ratios at which no cytotoxicity was detected from vehicle cultured cells or cells that had only seen DGKi at the time of priming (Fig. 5, E and F).

### DGKi synergizes with αPD1 therapy to control pancreatic and melanoma tumor growth

**Fig. 6.**
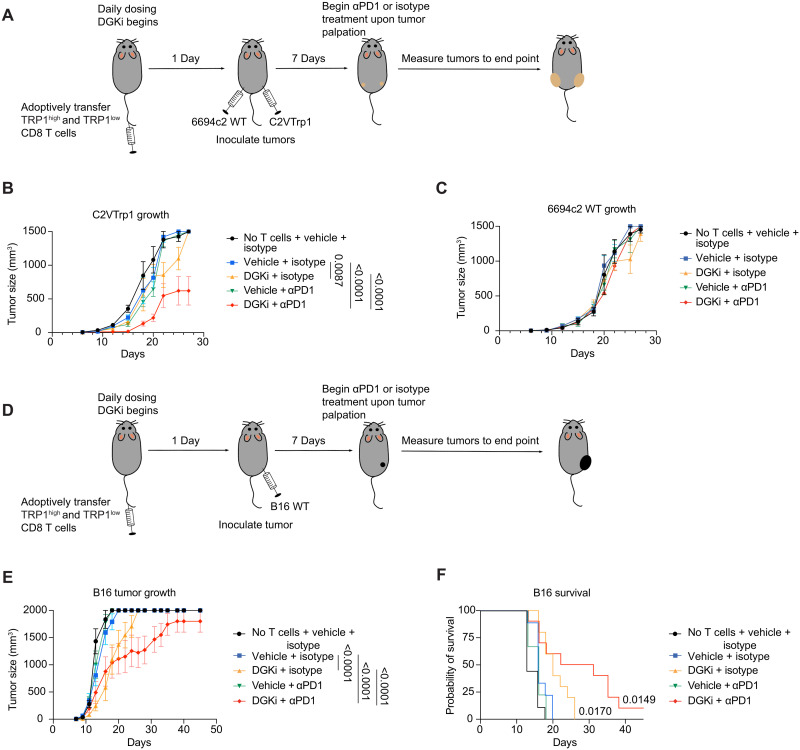
DGKi synergizes with αPD-1 therapy to control melanoma and pancreatic tumor growth in an antigen-dependent manner. (**A**) TRP1^high^ and TRP1^low^ CD8 T cells were isolated and adoptively transferred into C57BL/6 mice and dosed daily via oral gavage. The day after adoptive transfer, 6694c2 and C2VTrp1 tumors were inoculated on contralateral flanks. (**B**) C2VTrp1 and (**C**) 6694c2 tumor growth were measured until either tumor reached an endpoint tumor volume of 1500 mm^3^. Error bars represent SEM. (**D**) TRP1^high^ and TRP1^low^ CD8 T cells were adoptively transferred in C57BL/6 mice, and B16 tumors were inoculated the following day. (**E**) B16 tumor growth was measured until endpoint tumor volume of 2000 mm^3^. (**F**) Corresponding survival of mice was reported. Area under the curve for tumor growth was compared between groups via one-way ANOVA test and Tukey’s test for multiple comparison. Survival analysis was compared via a log-rank (Mantel-Cox) statistical comparison to vehicle + isotype group. Statistically significant *P* values are denoted.

To address whether DGKi could enhance antigen-specific antitumor immunity in vivo, we adoptively transferred TRP1^high^ and TRP1^low^ CD8 T cells into mice followed by inoculation of bilateral flank tumors of either C2VTrp1 or the parental 6694c2 cells (Fig. 6A). This mix of high- and low-affinity cells was used to model responses in humans spanning a range of TCR affinities. We observed a slight reduction in C2VTrp1 tumor growth in mice receiving TRP1 adoptive cell therapy compared to mice receiving no T cells, consistent with prior reports showing limited tumor control by TRP1 T cells in the absence of other therapies (*14*, *48*, *57*). Tumor-infiltrating CD8 T cells expressed high levels of PD-1, potentially restricting their therapeutic potential (fig. S8). The addition of DGKi or αPD-1 alone had no effect on these poorly immunogenic pancreatic tumors (*58*, *59*); however, combination therapy led to a significant decrease in C2VTrp1 tumor size (Fig. 6B). 6694c2 tumor growth was equivalent among all groups (Fig. 6C), indicating that endogenous T cell responses to 6694c2 cells are insufficient and that DGKi/αPD-1 enhances antigen-specific immunity by transferred TRP1 T cells.

We next tested whether DGKi could improve tumor control of TRP1^high^ and TRP1^low^ T cells against B16 melanoma, which expresses higher levels of Trp1 antigen. TRP1^high^ and TRP1^low^ CD8 T cells were adoptively transferred into C57BL/6 mice that were inoculated with B16 tumors (Fig. 6D). Treatment with adoptive cell therapy and αPD1 was insufficient to control tumor growth. TRP1 T cells combined with DGKi or DGKi/αPD-1 treatment further delayed tumor growth and improved median survival (Fig. 6, E and F).

### DGKi promotes TRP1^high^ and TRP1^low^ proliferation and cytokine production in mice with C2VTrp1 tumors

**Fig. 7.**
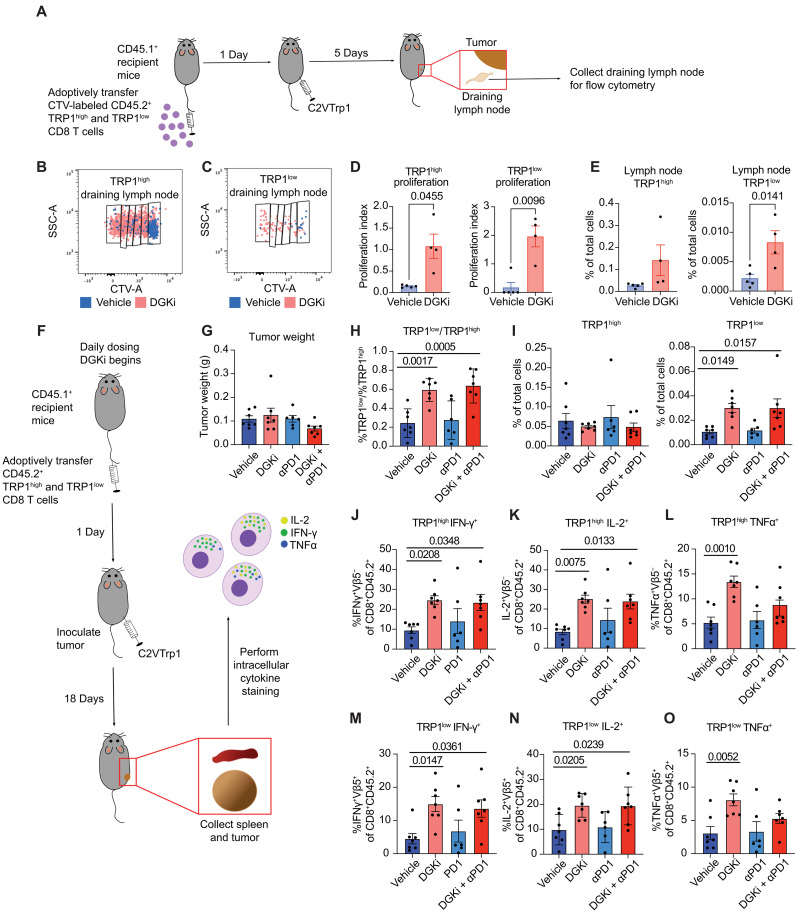
DGKi increases proliferation and cytokine production in antigen-specific T cells in mice bearing C2VTrp1 tumors. (**A**) CTV-labeled CD45.2^+^ TRP1^high^ and TRP1^low^ CD8 T cells were adoptively transferred in CD45.1^+^ recipient mice, and draining lymph node was collected 5 days after inoculation with C2VTrp1 cells. Representative flow plots of CTV staining for (**B**) TRP1^high^ and (**C**) TRP1^low^. (**D**) Proliferation indices of TRP1^high^ and TRP1^low^ CD8 T cells was quantified from CTV staining. (**E**) Fraction of TRP1^high^ and TRP1^low^ CD8 T cells from total cells in lymph node. (**F**) CD45.2^+^ TRP1^high^ and TRP1^low^ T cells were adoptively transferred in CD45.1^+^ recipient mice followed by inoculation of C2VTrp1 tumors the following day. Tumors were harvested 18 days later for intracellular cytokine staining for IFN-γ, TNFα, and IL-2. (**G**) C2VTrp1 tumor weights were measured after 18 days of growth in mice that received both TRP1^high^ and TRP1^low^ CD8 T cells. (**H**) The ratio of TRP1^low^ to TRP1^high^ CD8 T cells was determined from % CD8^+^CD45.2^+^TCRVβ5^+^ divided by the % CD8^+^CD45.2^+^TCRVβ5^−^ populations. (**I**) Fraction of TRP1^high^ and TRP1^low^ CD8 T cells from total cells in tumor. (**J** to **L**) TRP1^high^ or (**M** to **O**) TRP1^low^ producing IFN-γ, IL-2, and TNFα was determined from intracellular cytokine staining of C2VTrp1 tumors. Statistical comparisons were performed with one-way ANOVA with Dunnett’s test for multiple comparisons. Vehicle, DGKi, and DGKi + αPD1 groups: *n* = 7, αPD1: *n* = 6. Error bars represent SEM.

After observing a robust phenotype in DGKi-mediated T cell proliferation, cytokine production, and cytotoxic capacity in vitro, we asked whether DGK inhibition could facilitate increased proliferation and cytokine production in vivo. CTV-labeled CD45.2^+^ TRP1^high^ and TRP1^low^ CD8 T cells were adoptively transferred into CD45.1^+^ mice inoculated with C2VTrp1 tumors. The tumor-draining lymph node was harvested 5 days after tumor inoculation, and proliferation was measured via flow cytometry (Fig. 7A). TRP1^high^ and TRP1^low^ cells were distinguished by expression of different TCRVβs, as TRP1^low^ express Vβ5, whereas TRP1^high^ expressed Vβ8 (fig. S9). Both TRP1^high^ and TRP1^low^ T cells proliferated to a greater extent in the draining lymph node when mice were treated with DGKi (Fig. 7, B to D), resulting in increased total frequencies of TRP1-specific T cells (Fig. 7E). TRP1^high^ cells accumulated to a higher frequency than TRP1^low^ cells, potentially due to better engraftment of the TRP1^high^ cells after adoptive transfer or increased survival of TRP1^high^ cells upon exposure to antigen. Thus, DGKi has the capacity to increase priming of both TRP1^high^ and TRP1^low^ T cells in the presence of C2VTrp1 tumors in vivo.

To quantify cytokine production in vivo, CD45.2^+^ TRP1^high^ and TRP1^low^ CD8 T cells were adoptively transferred into C57BL/6 mice inoculated with C2VTrp1 tumors (Fig. 7F). Tumors and spleen were harvested 18 days later, when tumor weights had not significantly diverged (Fig. 7G). Although TRP1^high^ cells outnumbered TRP1^low^ cells in all groups, TRP1^low^ cells accumulated more in both groups treated with DGKi (Fig. 7, H and I). We found no significant difference in the accumulation of transferred TRP1^high^ cells in C2VTrp1 tumors among the treatment groups (Fig. 7I and fig. S10). We observed a greater frequency of intratumoral IL-2^+^, IFN-γ^+^, and TNFα^+^ producing TRP1^high^ and TRP1^low^ T cells in the groups treated with DGKi (Fig. 7, J to O, and fig. S10). This augmented cytokine production was only observed in the tumor; TRP1^high^ and TRP1^low^ T cells in the spleen produced negligible IL-2, IFN-γ, or TNFα (fig. S11), consistent with our findings that DGKi enhancement in cytokine production is highly antigen specific (Fig. 2, B to I). In addition, we observed decreased PD-1 expression in CD8 T cells and increased PD-L1 expression of tumor cells when DGKi was treated in combination with αPD-1 (fig. S8), justifying a combination therapy approach. Collectively, these studies demonstrate increased tumor-specific T cell proliferation and cytokine production upon DGKi treatment in vivo, with enhancements observed in both high- and low-affinity TCR clonotypes.

## DISCUSSION

Cancer immunotherapies have taken several approaches to enhancing antitumor immunity (*60*). Vaccines, oncolytic viruses, and innate adjuvants aim to activate dendritic cells and enhance T cell priming. IL-2 and its many variants support T cell survival and proliferation (*61*, *62*). Checkpoint blockade immunotherapies targeting PD-1/PD-L1, cytotoxic T lymphocyte–associated protein 4 (CTLA-4), or lymphocyte-activation gene 3 can reinvigorate T cells (*63*, *64*). All of these strategies rely on T cells to be able to recognize tumor antigenic peptides displayed on MHC-I. Chimeric antigen receptor T cells can circumvent this requirement for peptide-MHC recognition but as of yet have been ineffective against solid tumors in clinical settings.

Tumor antigenic peptides can arise from a variety of sources. Neoantigens arise from somatic mutations and viral antigens arise from tumors of viral origin such as human papillomavirus– or Epstein-Barr virus–associated tumors or Merkel cell carcinoma (*65*–*68*). Neoantigens and viral antigens are distinctly nonself, and T cells recognizing these antigens with high affinity can survive thymic selection (*69*). Other categories of tumor antigens include various versions of self-proteins that are expressed only in rare healthy tissues or only during gestation but are overexpressed in cancers or have altered posttranslational modifications (*12*). As one example, TRP1 is expressed by melanocytes as part of the melanin production pathway and is overexpressed in melanoma (*70*). Immune responses to TRP1 have been detected in both mice and human melanoma patients, and vaccination can further increase the abundance of TRP1-specific CD8 T cells (*70*, *71*). Although these findings suggest that some TRP1-specific CD8 T cells can escape negative selection in the thymus, presumably the highest affinity clonotypes do not. An analysis of TCR affinities between virus-specific and tumor self-antigen–specific clonotypes suggests that this affinity difference is broadly present (*19*). Low-affinity T cells can still contribute productively to anti-tumor immunity if properly activated, but how to activate these T cells in vivo and exploit this potential pool of anti-tumor T cells is unclear (*48*). Several approaches to augment expansion of low-affinity T cells have been reported including inhibition of Src homology region 2 domain–containing phosphatase-1 or depletion of T regulatory T cells (*72*, *73*). Critically, any such approaches must affect only antigen-specific T cells, as broader T cell activation could lead to autoimmune side effects (*74*, *75*).

Here, we take the novel approach of using DGKα/ζ inhibition to lower the threshold for TCR signaling. DGKα/ζ do not affect NFAT signaling, but restrict AP-1 and NF-κB signaling such that modest TCR signaling in cells with high DGKα/ζ activity promotes T cell unresponsiveness (*40*, *41*). Inhibition of DGKα/ζ leads to the opposite effect, namely, that modest TCR signaling leads to full T cell activation. Here, we have demonstrated enhancements in activation and effector function of two different T cell clones upon inhibition of DGKα/ζ activity. This enhancement was greatest in conditions where TCR stimulation was closest to the threshold between activation and nonactivation. TRP1^low^ T cell activation was consistently enhanced in the presence of DGKi as measured by proliferation and cytokine production. TRP1^high^ T cell activation with native Trp1 peptide was also enhanced as measured by cytokine production, but proliferation enhancements were only observed with suboptimal altered peptide ligands that did not induce maximal proliferation on their own.

TCR signaling is required for effector cells to engage target cells for cytolysis. Although minor differences in TCR signaling thresholds have been reported for effector cells versus naïve cells, both naïve and effector cell TCR signaling can be dampened by DGKα/ζ. Here, we demonstrate that DGKi is capable of lowering the threshold for TCR signaling in effector cells as shown in tumors with low antigen expression or low surface expression of MHC-I. Notably, TCR engagement of pMHC is still required as no cytolysis or cytokine production was observed during stimulation with β2m^−/−^ tumor cells that lack MHC-I completely. DGKi alone does not activate T cells; antigen engagement is still required. This distinction bodes well for use of DGKi therapeutically where autoimmunity remains a significant concern for patients receiving checkpoint blockade immunotherapy. However, tonic signaling via self-peptide MHC complexes may still lead to a burst of T cell activation upon treatment with DGKi. Use of small-molecule cellular inhibitor of apoptosis proteins 1/2 antagonists mimics costimulatory signals in T cells and similarly requires TCR engagement of cognate pMHC complexes (*76*). Nevertheless, use of these inhibitors clinically results in transient increases in serum cytokines (*77*). Organ-specific autoimmunity is common with monoclonal antibody blockade of CTLA-4 or PD-1/PD-L1 pathways (*75*). These inflammatory side effects can affect any organ but are more prevalent in barrier-exposed tissues where resident memory T cells can be converted to proliferating, IFN-γ–producing cytotoxic T cells (*75*, *78*). To what extent DGKi will result in activation of polyclonal or self-reactive T cells in humans remains to be determined.

Defects in MHC-I antigen presentation are a major mechanism of both primary and acquired resistance to immunotherapy (*26*). Although loss of β2m in tumors eliminates expression of MHC-I, other alterations such as loss of IFN-γ signaling or defects in antigen presentation pathway can lead to reduced but not absent MHC-I levels and consequently loss of response to PD-1/PD-L1 blockade (*54*, *79*). We have modeled low expression of pMHC in vitro and in vivo and shown that DGKi can augment cytotoxicity in settings of sparse antigen density or diminished MHC-I expression. In vivo, DGKi combined with PD-1 blockade slowed tumor growth in settings where PD-1 blockade alone was ineffective, suggesting that DGKi could expand the range of patients who could benefit from PD-1/PD-L1 blockade.

Collectively, our data demonstrate the profound impact of inhibition of DGKα/ζ activity in antigen-specific T cells. DGKα/ζ inhibition augments CD8 T cell priming and cytolytic function in vitro as well as in vivo. Mechanistically, DGKi lowers the threshold for TCR signaling, thereby recruiting lower-affinity antitumor T cells as well as higher affinity clonotypes. This broadening of the TCR repertoire is also accompanied by increased production of IL-2, IFN-γ, and TNFα even in settings of low MHC-I where checkpoint blockade alone is ineffective.

## MATERIALS AND METHODS

### Study design

Previous work from our laboratory generated the TRP1^high^ and TRP1^low^ CD8 T cells, and binding to Trp1 peptide:MHC tetramers and activation and effector functionality were characterized. Our primary objective was to investigate whether inhibition of DGKα/ζ could augment activation and effector function of higher- and lower-affinity TCRs both in vitro and in vivo. To evaluate activation, we performed CD8 T cell priming assays by coculturing peptide-pulsed antigen-presenting cells with CD8 T cells with four biological replicates per condition. Proliferation, IL-2 and IFN-γ production were quantified to evaluate overall CD8 T cell activation. TRP1^high^ and TRP1^low^ killing of tumor cell lines was assessed using zsGreen-expressing B16, 6694c2, C2VTrp1, and C2VTrp1 β2m^−/−^ cells with six to eight biological replicates per condition. DGKi in vivo efficacy was assessed in subcutaneous B16 and C2VTrp1 tumor models with 5 to 10 mice enrolled, as denoted in the figure legends. Proliferation and cytokine staining of TRP1^high^ and TRP1^low^ T cells were assessed in subcutaneous C2VTrp1 tumor models with four to seven mice enrolled per group, as denoted in the figure legends. All mice were randomized before study enrollment.

### Mice

All animal protocols were approved by the Dana-Farber Cancer Institute Committee on Animal Care (protocol nos. 14-019 and 14-037) and are in compliance with the National Institutes of Health (NIH)/National Cancer Institute ethical guidelines for tumor-bearing animals. The following mouse strains were purchased from the Jackson Laboratory: C57BL/6J (stock no. 000664), Myc-GFP (B6;129-Myctm1Slek, stock no. 021935), RAG2^−/−^ [B6(Cg)-Rag2tm1.1Cgn/J, stock no. 008449], IFN-γ^−/−^ (B6.129S7-Ifngtm1Ts/J stock no. 002287), Prf1^−/−^ (C57BL/6-Prf1tm1Sdz/J, stock no. 002407), OT-I [C57BL/6-Tg(TcraTcrb)1100Mjb/J, stock no. 003831], and CD45.1^+^ (B6.SJL-Ptprca Pepcb/BoyJ, stock no. 002014). TRP1^high^ (stock no. 030958) and TRP1^low^ (stock no. 030957) mice were generated by us as previously described and bred in-house (*48*).

### CD8 T cell isolation and culture conditions

CD8 T cells were obtained from spleen and inguinal lymph nodes and mechanically homogenized through 40-μm filters. CD8 T cells were isolated via magnetic bead enrichment with an EasySep Mouse CD8^+^ T cell isolation kit (STEMCELL Technologies, catalog no. 19853). CD8 T cells were cultured in 2 ml per well in a 12-well plate with RPMI complete media composed of RPMI 1640 media (Life Technologies, 11875119) supplemented with 10% fetal bovine serum, 1 mM sodium pyruvate (Gibco, catalog no. 11360070), 2 mM GlutaMAX (Gibco, catalog no.35050061), 0.1 mM nonessential amino acids (Gibco, catalog no. 11140050), penicillin/streptomycin (100 U/ml; Gibco, catalog no. 15140122), and 0.11 M β-mercaptoethanol (Sigma-Aldrich, M3148).

### T cell receptor signaling and Western blot analysis

Effector T cells differentiated for cytotoxicity assays or naïve CD8 T cells were plated on agonistic anti-mouse CD3 and CD28 (BioLegend, 100340) for 30 to 60 min. T cells were harvested and lysed in lysis buffer composed of 50 mM Hepes, 50 mM NaCl, 0.5% NP-40 with protease, and phosphatase inhibitors in deionized water. Bicinchoninic acid assay was performed to determine concentration of lysates. Equal amounts of cell lysates were loaded in 4 to 20% SDS–polyacrylamide gel electrophoresis gels followed by a transfer to a polyvinylidene difluoride (PVDF) membrane using Bio-Rad’s Trans-blot Turbo Transfer System. Detection antibodies were incubated in 3% BSA in TBST buffer composed of tris buffer, NaCl, and Tween 20. The following antibodies were used for detection of target proteins: phospho-ERK1/2 (ab201015), total-ERK1/2 (ab184699), phospho-AKT(S473) (clone D9E, CST 4060S), phosphor-AKT(T308) (clone D25E6, CST 13038), total AKT (CST 9272S), and NF-κB inhibitor α (IκBα) (CST 4812).

### CD8 T cell priming assay

Antigen-presenting cells were isolated from C57BL/6 splenocytes. Briefly, spleen was collected and mechanically homogenized through 40-μm filters, and B cells were isolated via negative selection with mouse CD43 magnetic Dynabeads (untouched B cells) (Life Technologies, 11422D) and activated with anti-mouse CD40 agonist (clone HM40-3) for 2 days. On day 2, antigen-presenting cells were pulsed with peptide of interest as previously described (*14*). Concurrently, CD8 T cells (TRP1^high^, TRP1^low^, or OT-I) were isolated from the spleen and inguinal lymph nodes, and CD8^+^ T cells were isolated using the EasySep Mouse CD8^+^ T cell isolation kit (STEMCELL Technologies, catalog no.19853). CD8 T cells were stained with CTV (Life Technologies, catalog no. C34557) following isolation as per the manufacturer’s protocol. T cells and antigen-presenting cells were cocultured in a 96-well U-bottom plate at a 2:1 T:B cell ratio in RPMI complete media supplemented with human IL-2 (100 U/ml) (PeproTech, catalog. no. 200-02). Proliferation was quantified via a proliferation index representing the fraction of mitotic events by the number of progenitor cells. The mathematical expression for this index is as follows

$$\text{Proliferation index}=\frac{\sum_{i=1}^{n}D_{i}}{\sum_{i=0}^{i=n}\frac{1}{2^{i}}*D_{i}}$$

where *i* represents division number and *n* represents maximum division number.

### IL2, IFN-γ, and ΤΝFα ELISAs

Mouse cytokine levels for IL2, IFN-γ, and TNFα were quantified using BioLegend’s enzyme-linked immunosorbent assay (ELISA) MAX Deluxe set for the respective cytokine (IL-2, catalog no. 431004; IFN-γ catalog no. 430804; and TNFα, catalog no. 430904). Manufacturer’s protocol was followed with modification to development stage. Development was conducted with 100 μl of 3,3′,5,5′-tetramethylbenzidine (Sigma-Aldrich catalog no. T0440-1L) and development was terminated with 50 μl of 1 M HCl. Absorbance at 450 nm was read using an Envision Plate Reader (PerkinElmer).

### TRP1^high^ x Myc-GFP T cell priming assay

TRP1^high^;Myc-GFP CD8 T cells were isolated and stained with CTV as described above and plated on αCD3/28 (1 μg/ml)–coated 24-well plates. After 24, 48, and 72 hours, cells and supernatants were separated and collected by centrifugation. Flow cytometry was used in determining proliferation and Myc expression via GFP signal.

### C2VTrp1 cell line generation

The pEF1a_IRES_zsgreen was the backbone plasmid used to introduce the *Tyrp1* gene into the pancreatic cancer cell line 6694c2 (a gift from B. Stanger, University of Pennsylvania) (*59*). Briefly, mouse *Tryp1* was inserted into the vector at cut sites Eco RI (NEB, R3101L) and Bam HI (NEB, R3136L). 6694c2 cells were plated in a six-well plate 48 hours before transfection and achieved a confluency of about 80% on the day of transfection. Two micrograms of vector (empty) or 2.77 μg of vector (Tyrp1-containing gene) was mixed with 2 μl of Lipofectamine (Life Technologies, catalog no. 11668027) in Opti-MEM media (Life Technologies, catalog no. 31985062) and allowed to incubate for 15 min before dropwise addition to cells. Cells were incubated for 24 hours followed by replenishment with new RPMI media. Forty-eight hours after transfection, cells were provided RPMI complete containing the selection agent Geneticin (Gibco, catalog no. 11558616) at 50 mg/ml. Selection was performed for 2 weeks with daily removal and replenishment of RPMI complete + Geneticin. After 2 weeks, cells were sorted (BD Aria II) for zsgreen expression. Successful Tyrp1 protein expression was validated through Western blot. Briefly, C2VTrp1, 6694c2 with empty vector, and B16 WT cells were lysed in lysis buffer (see T cell receptor signaling and Western blot analysis). Protein quantities were assessed via bicinchoninic acid assay (Thermo Fisher Scientific, catalog no. 23235), and 45 μg of lysate was loaded on a 4 to 20% polyacrylamide gel (Bio-Rad, catalog no.5671093) and transferred to a PVDF membrane using Bio-Rad’s Trans-blot Turbo Transfer System. Tyrp1 was blotted at 5 μg/ml of Tyrp1 antibody and β-actin–horseradish peroxidase was blotted (dilution 1:3000) to serve as a loading control.

### CD8 T cell cytotoxicity assay of tumor cells

CD8 T cells were isolated as described above. T cells were then activated with mouse CD3/28 Dynabeads (Life Technologies, catalog no. 11452D) and hIL2 (100 U/ml) in media in a 12-well plate for 48 hours. On day 2, magnetic CD3/28 beads were removed, and CD8 T cells were reconstituted in fresh RPMI media supplemented with hIL2. To facilitate CD8 T cell differentiation into cytotoxic effectors, T cells were split every 2 days and supplemented with fresh media and hIL2 until days 7 and 8. One day before coculture between T cells and tumor cells, zsgreen-expressing tumor cells were plated on a 96-well plate unless otherwise stated in the presence or absence of IFN-γ (10 ng/ml). The next day, the number of tumor cells was counted using a plate-based imaging cytometer (Celigo). Confluence was measured via the Celigo instrument using zsgreen expression at various time points between 6 and 48 hours. The average confluence from the “tumor-cells only” condition was used as the reference value for calculating the percent cytotoxicity. The quantitative values were obtained using the following confluence values: $\%\mathrm{Cytotoxicity}=\frac{\bar{\mathrm{Tumor}\;\mathrm{only}}-\mathrm{condition}}{\bar{\text{Tumor only}}}$. Supernatants were collected at the final time point for analysis of IL-2, IFN-γ, and TNFα production via ELISA.

### IL-2, IFN-γ, and TNFα transcript levels during priming and cytotoxicity

CTV-labeled TRP1^high^ CD8 T cells were activated by B cells treated with native Trp1 peptides (500 pg/ml) (±100 nM DGKi) for 24, 48, and 72 hours following the protocol described above. A fraction of cells was collected to assess proliferation by CTV expression. The other fraction of cells was lysed for RNA isolation with Qiagen’s RNeasy Plus mini kit (catalog no. 74134) as per the manufacturer’s protocol. For cytotoxicity assay, effector Trp1hi CD8 T cells were cocultured with C2VTrp1s in a 24-well plate for 24 hours. Supernatants and cells were collected at indicated time points. Protein cytokine levels were quantified as described above via ELISA, and RNA was isolated for quantitative polymerase chain reaction.

### DGKi in vitro and in vivo formulation and dosing

The DGKi inhibitor was provided by Bristol Myers Squibb (BMS) and was reconstituted in dimethyl sulfoxide at a concentration of 20 mM for in vitro experiments. For in vivo formulation and dosing, the vehicle solution was prepared at a 90:5:5 weight ratio of polyethlyene glycol 400 (Fisher Scientific, AAB219920B), d-α-tocopherol polyethylene glycol 1000 succinate (Sigma-Aldrich 57448), and 200 proof ethanol (Fisher Scientific, 07678004). DGKi was added at a final concentration of 60 μg/150 μl, and mice were orally gavaged with 150 μl of DGKi or vehicle daily. Antibodies to PD-1 (clone no. RMP1-14) or isotype control (clone no. LTF-2) were obtained from Bio X cell and were administered to mice by intraperitoneal injection at 150 mg per mouse every 3 days.

### Subcutaneous tumor inoculations

TRP1^high^ and TRP1^low^ CD8 T cells were isolated and prepared for adoptive transfer as described above. C57BL/6 mice were irradiated with 100 rad, and 6 hours later, approximately 1 million TRP1^high^ and 1 million TRP1^low^ cells were injected intravenously into recipient mice. The next day, 250,000 C2VTrp1 and 250,000 6694c2 tumor cells were injected subcutaneously on opposite flanks in endotoxin-free PBS. Mice received daily dosing of DGKi via oral gavage, and αPD1 or isotype treatment began upon the detection of palpable tumors and dosed every 3 days. Tumor measurements were made every 2 to 3 days, and mice were euthanized when either tumor reached 2000 mm^3^, ulcerated, or if mice met humane endpoints. B16-F10 cells were obtained from the American Type Culture Collection and were inoculated subcutaneously at 250,000 cells per mouse. Mice were treated and monitored as described above. All tumor cells tested negative for mouse pathogens and were screened for mycoplasma every 3 to 4 months throughout the study.

### TRP1^high^ and TRP1^low^ priming in mice bearing C2VTrp1 tumors

Spleen and lymph nodes were collected from TRP1^high^ mice and TRP1^low^;Rag2^−/−^ mice, and CD8 T cells were isolated by magnetic bead separation. Upon isolation, TRP1^high^ and TRP1^low^ cells were counted, stained for CTV per the manufacturer’s protocol (Life Technologies, catalog no. C34557), and combined in endotoxin-free PBS (Thermo Fisher Scientific, catalog no. TMS012A). C57BL/6 mice were irradiated with 100 rad and 6 hours later received tail vein injections of approximately 2 million TRP1^high^ and 1 million TRP1^low^ T cells. The following day, mice were inoculated with 250,000 C2VTrp1 tumor cells in endotoxin-free PBS. Five days after tumor inoculation, mice were euthanized, and the draining inguinal lymph node was collected. Lymph nodes were digested as follows. Each lymph node was placed and pierced with forceps in RPMI 1640 media supplemented with Dispase II (0.8 mg/ml; Life Technologies, 17105041), collagenase P (0.2 mg/ml; Sigma-Aldrich, 11249002001), and deoxyribonuclease I (DNase I) (0.1 mg/ml; Sigma-Aldrich, 10104159001). Lymph nodes were incubated in this media for 30 min at 37°C. After complete digestion, the solution containing Dispase II, collagenase P, and DNase I was neutralized with fluorescence-activated cell sorting buffer containing 2% fetal bovine serum and 2 mM EDTA in PBS. Cells were then stained with CD8 BV785 (BioLegend, 100750), CD45.2 fluorescein isothiocyanate (BioLegend, 109806), TCRVβ5 allophycocyanin (APC; BioLegend, 139505), and TCRVβ8 phycoerythrin (PE) (BioLegend, 140104), and samples were read using Sony SP6800 spectral flow cytometer.

### Intracellular cytokine staining for IL2, IFN-γ, and TNFα in mice bearing C2VTrp1 tumors

TRP1^high^ and TRP1^low^ CD8 T cells were isolated and prepared for adoptive transfer and the manner described above. C57BL/6 mice were irradiated with 100 rad and 6 hours later received tail vein injections of approximately 1 million TRP1^high^ and 1 million TRP1^low^ T cells. After 1 day, mice were inoculated with 250,000 C2VTrp1 cells in endotoxin-free PBS. Mice were euthanized, and spleen and tumor were collected 18 days after tumor inoculation. C2VTrp1 tumors were chopped and digested in RPMI 1640 media supplemented with soybean trypsin inhibitor (concentration) (Life Technologies, 17075029) and collagenase (concentration) for 30 min at 37°C. Next, tumor fragments were mixed with a pipette, and the solution was transferred to a 5-ml flow tube. Undigested tumor fragments were replenished with fresh digestion media and incubated for 30 min at 37°C. The samples were then remixed and transferred to their respective 5-ml flow tube. Spleens were mashed through a 40-μm filter, and ammonium chloride potassium (ACK) lysis was performed and neutralized with PBS. Intracellular cytokine staining was performed as follows: Tumor and spleen samples were stained for extracellular markers CD8a BV785 (BioLegend, 100750), CD45.2 PE (BioLegend, 109807), and TCRVβ5 APC (BioLegend, 139505). Cells were fixed in fixation buffer (BioLegend, 420801) for 20 min at room temperature in the dark. Cells were washed in permeabilization/wash buffer (BioLegend, 421002) twice and then stained for cytokines, IL-2 BV711 (BioLegend, 503837), IFN-γ BV421 (BioLegend, 505830), and TNFα PE/Cy7 (BioLegend, 506323) overnight at 4°C. The next day, samples were washed in Perm/Wash Buffer and samples were read on a Sony SP6800 spectral flow cytometer.

### Statistical analysis

All statistical analysis was performed in GraphPad Prism. In vitro experiments were presented with means and SEM error bars unless otherwise noted in figure legends. Analysis of variance (ANOVA) with Sidák’s test for multiple comparisons was performed for three groups or more. All tumor and tumor infiltrate data were presented as means and SEM error bars. Statistical analysis of tumor growth curves was performed with ANOVA and Dunnett’s test for multiple comparisons. Survival was compared via a log-rank (Mantel-Cox) test. Analysis of proliferation and cytokine production of tumor-infiltrating CD8 T cells was compared with a one-way ANOVA with Dunnett’s test for multiple comparisons. Numerical *P* values are denoted on all figure panels.
