# Supplementary material for: DGKα/ζ inhibition lowers the TCR affinity threshold and potentiates antitumor immunity
Source: Sci Adv. 2023 Nov 24;9(47):eadk1853. doi: 10.1126/sciadv.adk1853 (PMC10672170; doi:10.1126/sciadv.adk1853)
Supplement: Supplementary file 1 — Figs. S1 to S11 [file sciadv.adk1853_sm.pdf]

Science Advances
AAAS

Supplementary Materials for

DGKα/ζ inhibition lowers the TCR affinity threshold and potentiates antitumor immunity

Rakeeb Kureshi *et al.*

Corresponding author: Stephanie K. Dougan, stephanie_dougan@dfci.harvard.edu

Sci. Adv. **9**, eadk1853 (2023)
DOI: 10.1126/sciadv.adk1853

This PDF file includes:

Figs. S1 to S11

Supplementary Figures

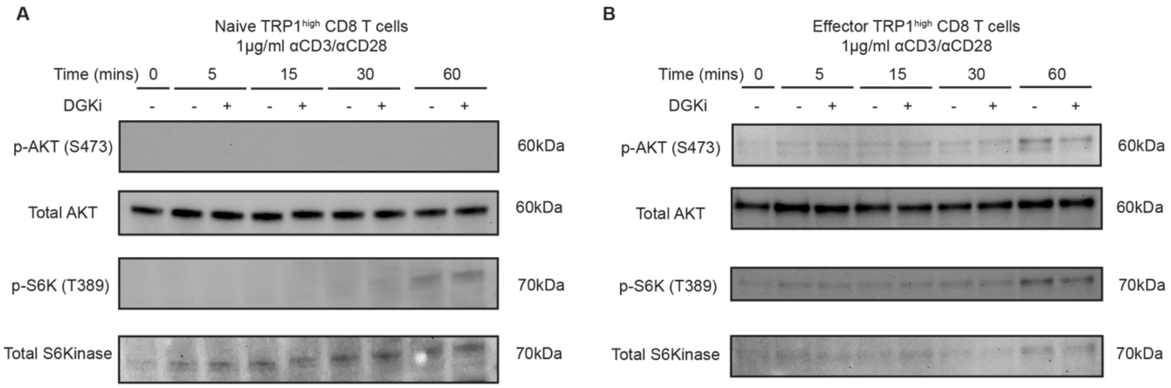

Fig. S1. DGKi treatment does not impact MTORC-1/2 activity at early time points. (A) Unactivated or (B) Effector TRP1^{high} CD8 T cells were activated with αCD3/αCD28 for 5, 15, 30, and 60 minutes with or without DGKi. No phospho-AKT was detected for naïve TRP1^{high} and p-AKT is the lower band for effector TRP1^{high}. Immunoblot analysis was conducted for the phosphorylation of S6 Kinase (T389) and AKT (S473) to assess MTORC-1 and -2 activities respectively.

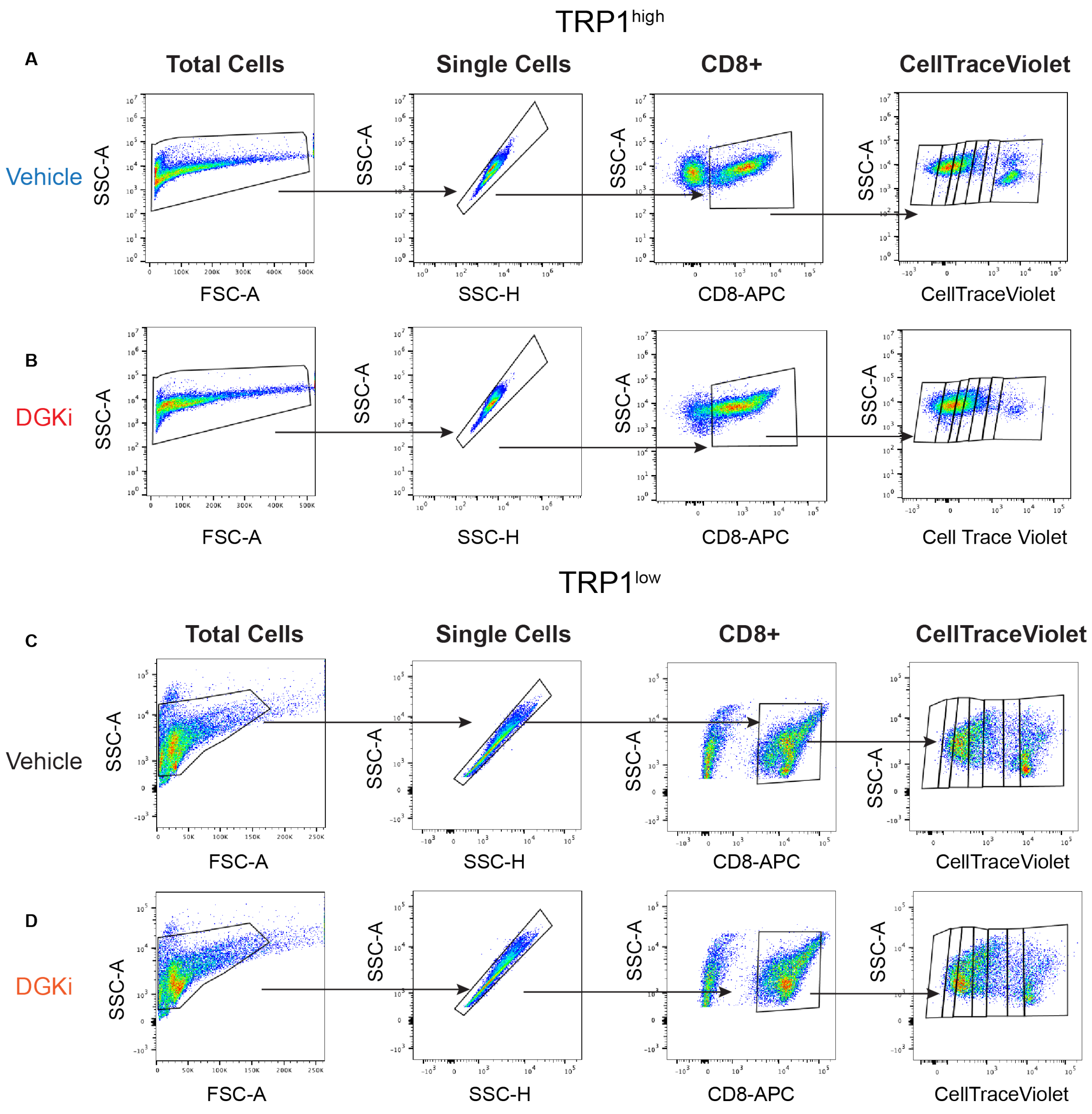

Fig. S2. Representative flow plot for T cell priming and proliferation analysis. General gating scheme for CellTraceViolet-labeled (A-B) TRP1^{high} or (C-D) TRP1^{low} CD8 T cells activated in the presence of B cells pulsed with 50pg/ml native Trp1 peptides +/- 150 nM DGKi.

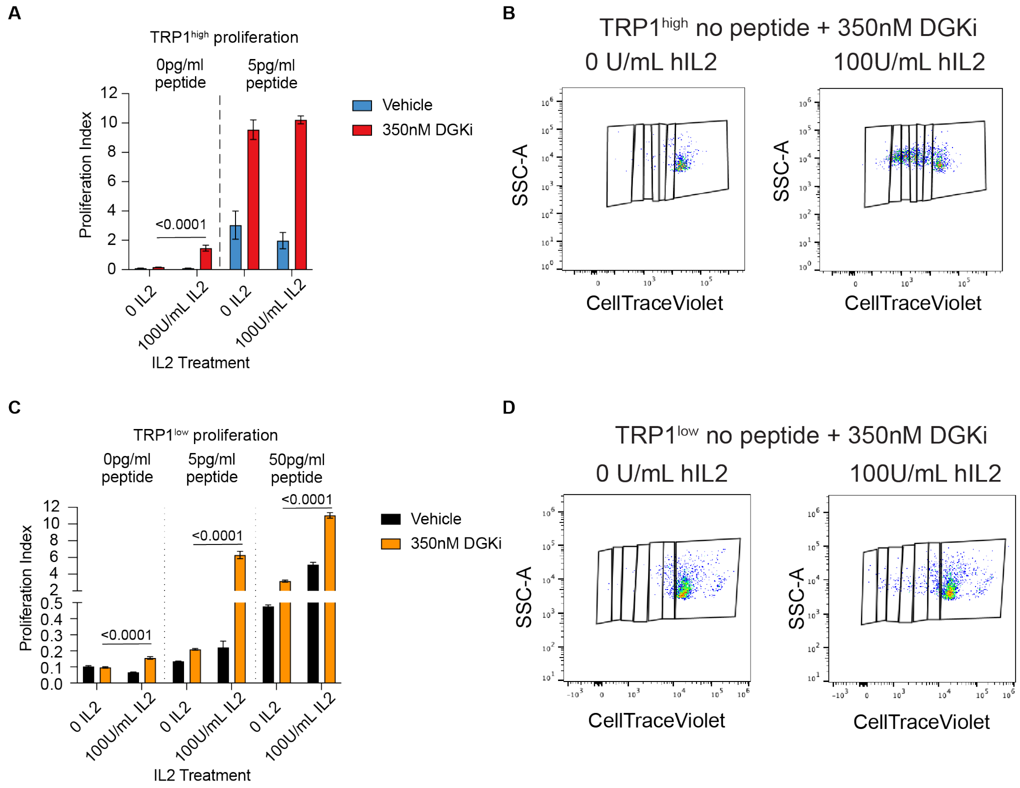

Fig. S3. IL-2 augments DGKi-mediated proliferation. CTV-labeled (A) TRP1^{high} or (C) TRP1^{low} CD8 T cells were activated with B cells and native Trp1 peptide for 84 hours +/- 100U/mL hIL-2 and/or 350nM DGKi. Representative flow plots for (B) TRP1^{high} or (D) TRP1^{low} cultured with B cells in the absence of peptide, 350nM DGKi, and denoted concentrations of hIL-2 are shown. Error bars represent SEM (n = 4). Statistical comparisons were conducted within each peptide concentration with a two-way ANOVA and Sidak's test for multiple comparisons.

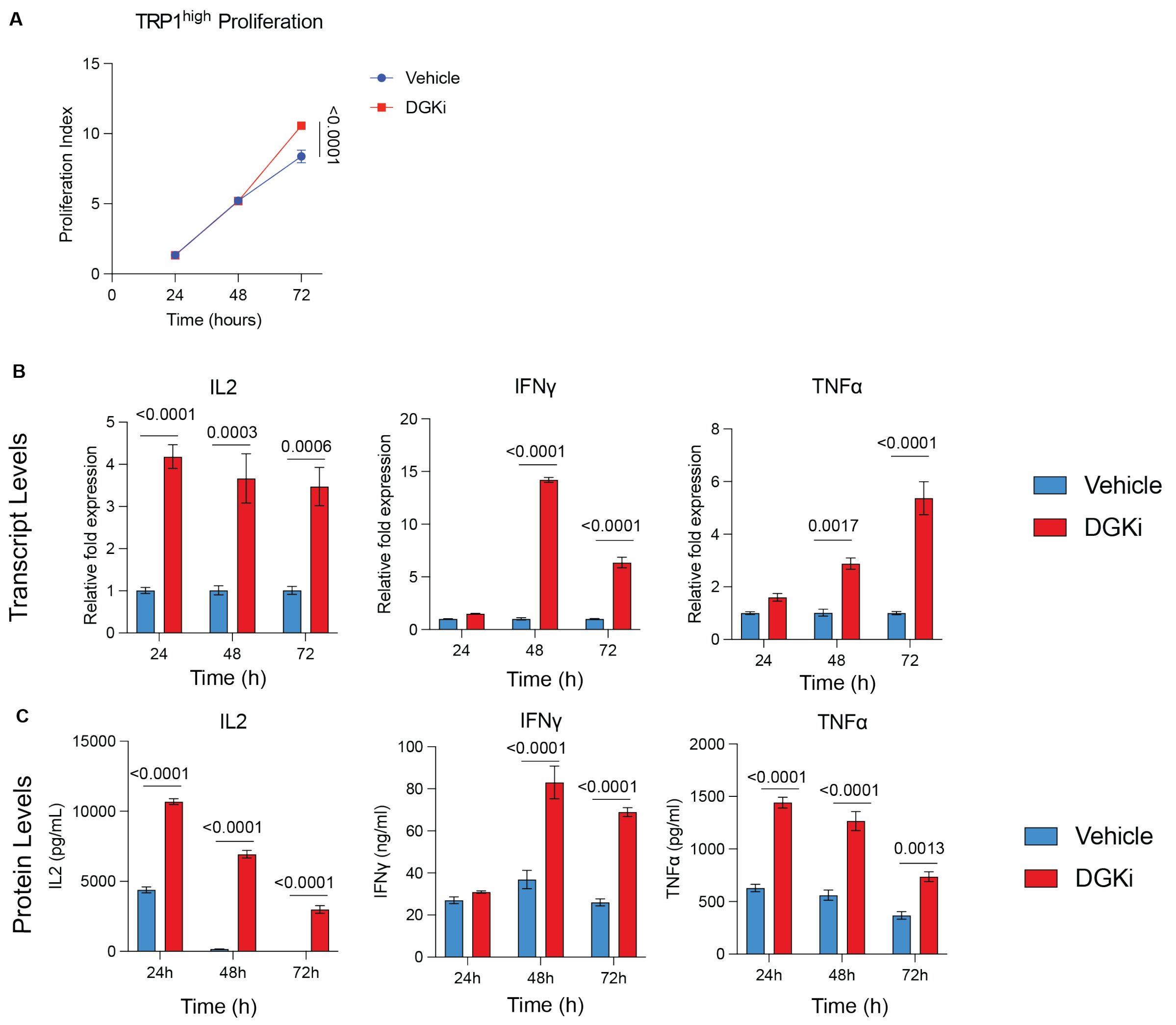

Fig. S4. DGKi upregulates cytokine transcript and protein expression during T cell priming. (A) TRP1^{high} CD8 T cells were cocultured with B cells pulsed with 500 pg/ml native Trp1 peptide and proliferation was determined via CTV staining at indicated time points. (B) Cells were also collected for RNA analysis via qPCR and (C) supernatants were collected to determine IL2, IFNγ, and TNFα proteins levels via ELISA at indicated time points (n = 3 for each treatment and time point). Error bars represent SEM. Transcript and protein levels were compared with Two-way ANOVA and Sidak's test for multiple comparisons. Statistically significant P-values are denoted above compared groups.

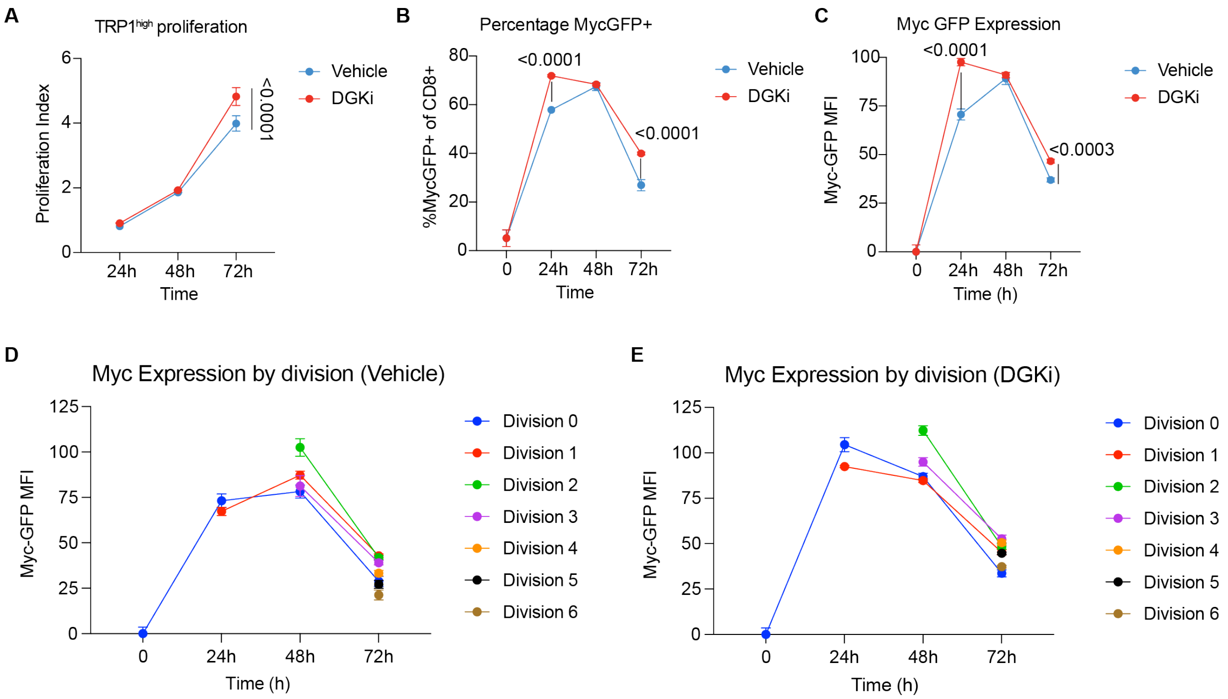

Fig. S5. DGKi upregulates Myc expression in TRP1^{high} CD8 T cells in a temporal manner. CTV-labeled TRP1^{high} CD8 T cells expressing Myc-GFP were activated with 1μg/ml αCD3/28 coated plates for 24, 48, and 72 hours. (A) Proliferation Index was quantified via CTV expression. Myc expression was quantified through (B) percentage of CD8 cells expressing Myc and (C) magnitude of Myc quantified via mean fluorescence intensity. Myc MFI was quantified for every division from CTV intensity for (D) vehicle and (E) DGKi treated TRP1^{high} CD8 T cells (n = 3 for all groups). Error bars represent SD. Statistical comparisons were conducted with a Two-way ANOVA and Dunnett's test for multiple comparisons to the vehicle control group. Statistically significant P-values are denoted above compared groups.

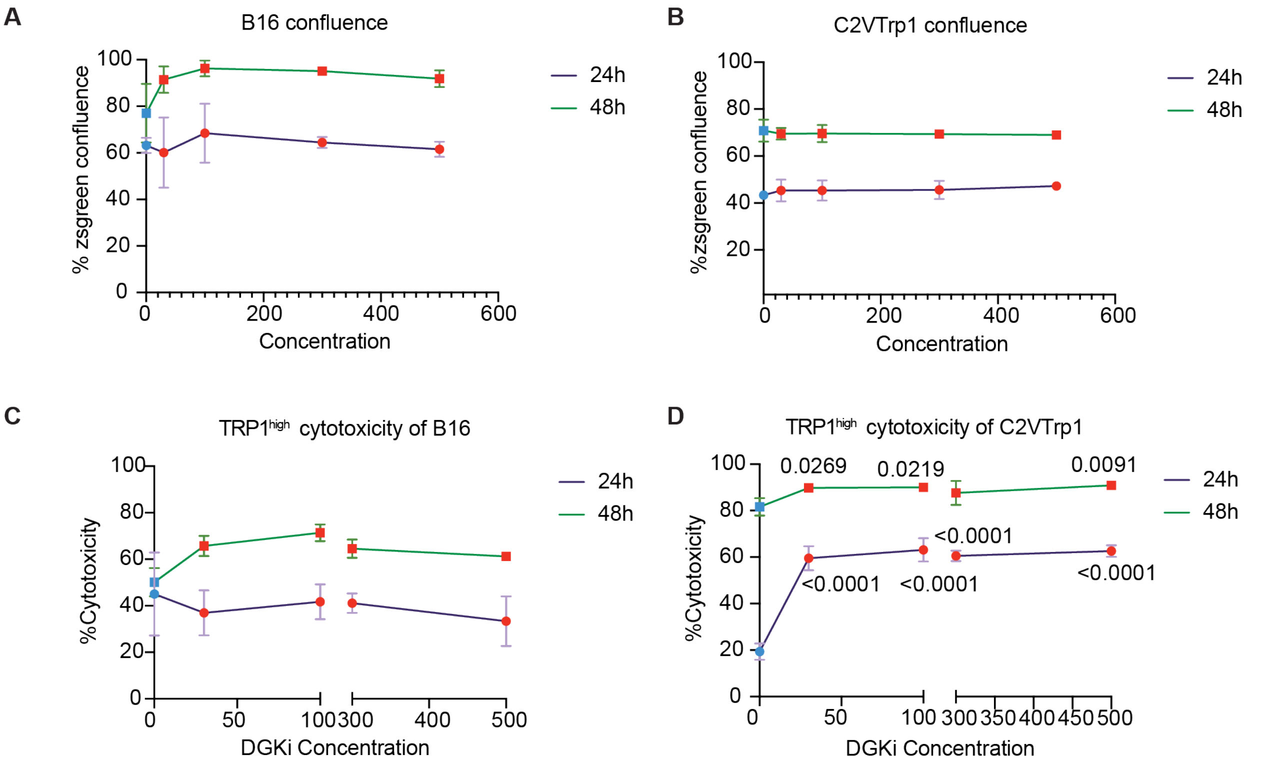

Fig. S6. Tumor growth is unaffected by DGKi in the absence of antigen-specific T cells. B16 zsgreen or C2VTrp1 Tumor cells were incubated with and without TRP1^{high} CD8 T cells and increasing concentrations of DGKi for 24 and 48 hours. Zsgreen confluency was measured for (A) B16 and (C) C2VTrp1 tumor only wells with the Celigo Imaging Cytometer. TRP1^{high} CD8 T cells were cocultured with (C) B16 or (D) C2VTrp1 cells for 24 to 48 hours at varying concentrations of DGKi and %cytotoxicity was calculated from zsgreen confluency (n = 4). Error bars represent SEM. Confluency and cytotoxicity was compared with a Two-way ANOVA with Sidák's test for multiple comparisons. Statistically significant P values are denoted between vehicle (0 nM) and DGKi treated conditions.

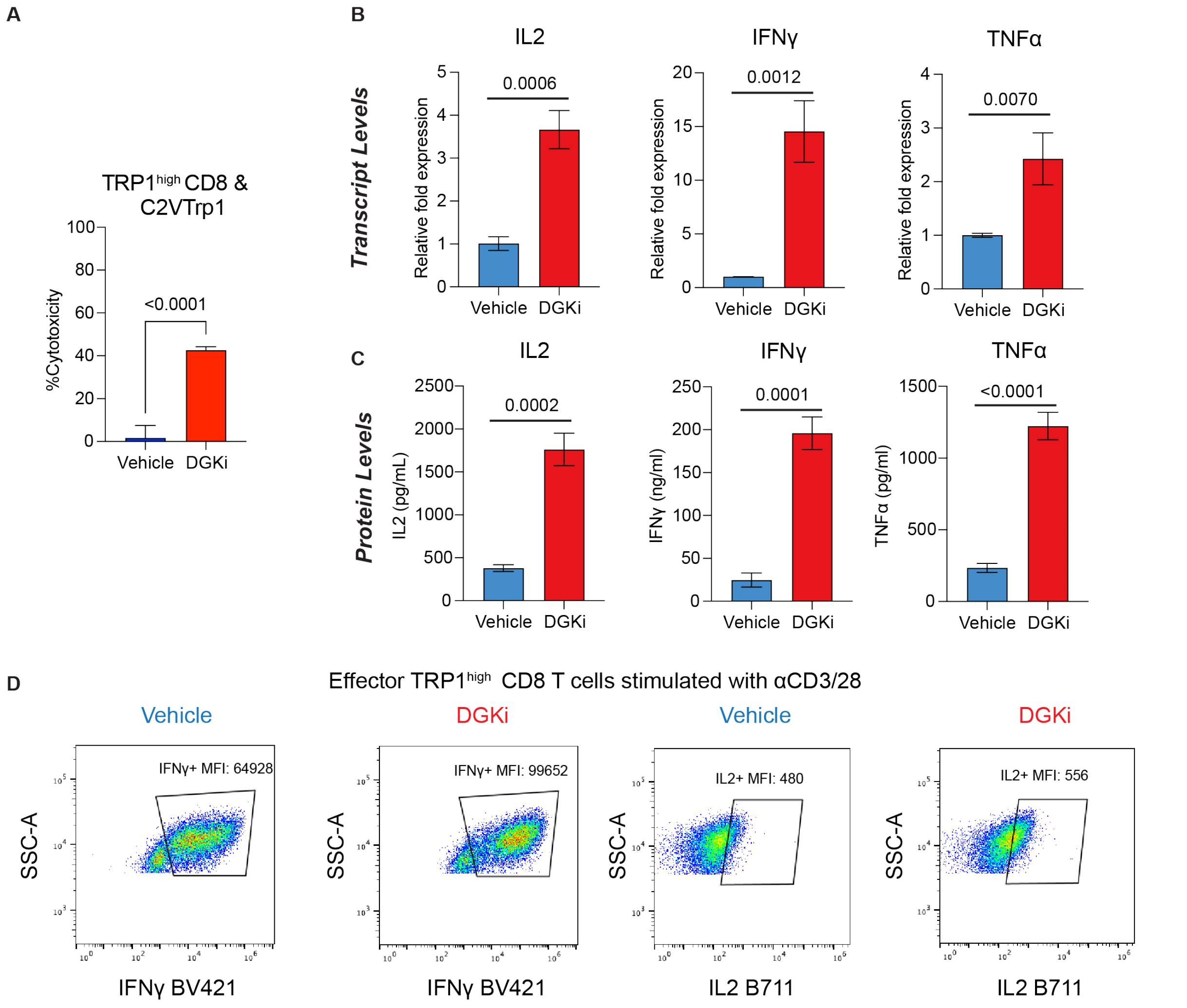

Fig. S7. DGKi enhances CD8 T cell cytokine transcript levels and protein expression during coculture with tumor cells. (A) TRP1^{high} CD8 T cells were cocultured with C2VTrp1 tumor cells for 24 hours in the presence or absence of 100nM DGKi. (B) Cells were collected for RNA isolation and analysis. Transcript levels of IL2, IFNγ, and TNFα was determined by qPCR. (C) Supernatants were collected and ELISAs were performed to determine protein levels of the aforementioned three cytokines. Comparisons of cytotoxicity, transcript, and protein levels were quantified with student's t-test. (D) Effector TRP1^{high} CD8 T cells were activated with 1µg/ml αCD3/28 (+/- 100nM DGKi) for 24 hours followed by assessment of IFNγ and IL2 production by intracellular cytokine staining.

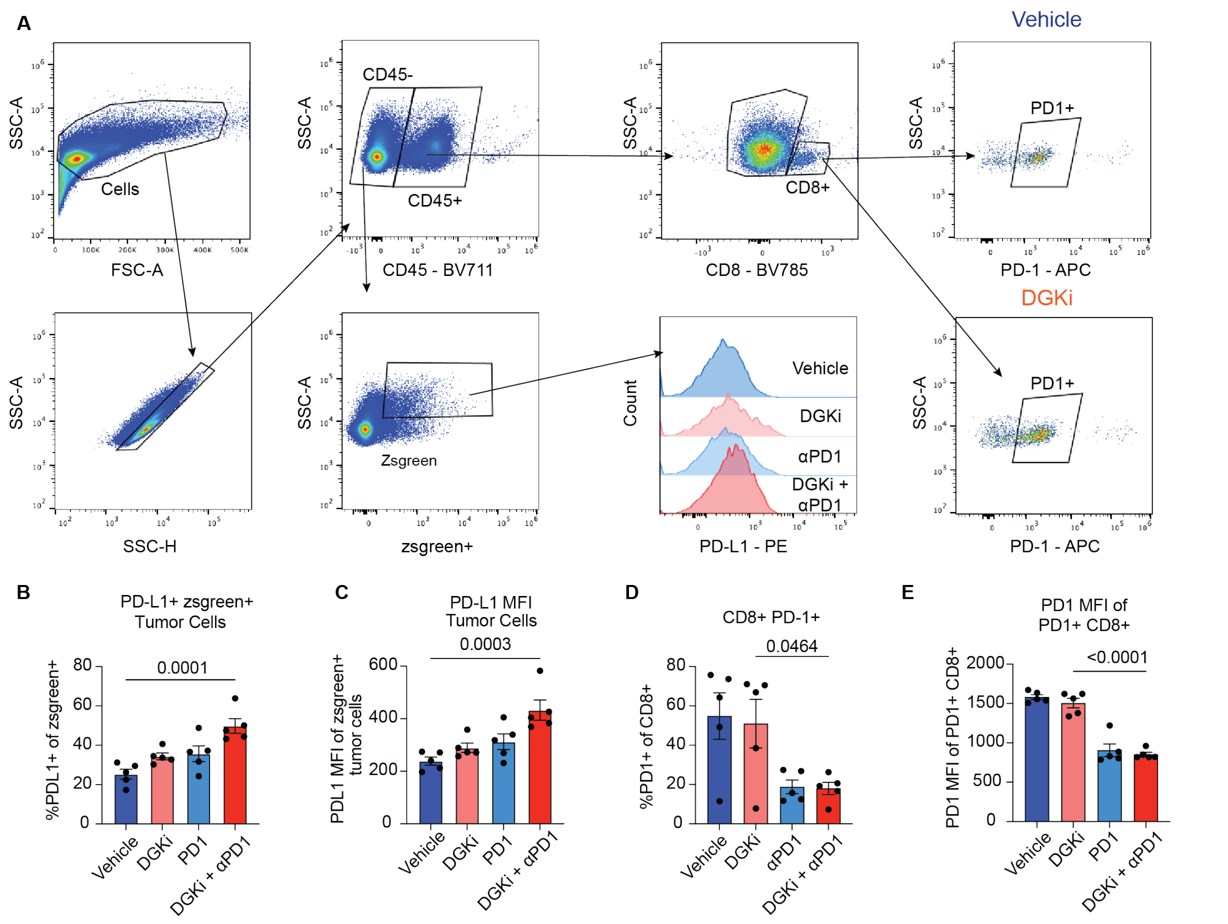

Fig. S8. DGKi and αPD1 in combination decreases CD8 T cell PD-1 expression and increases tumor cell PD-L1 expression. TRP1^{high} and TRP1^{low} were adoptively transferred into irradiated C57BL/6 mice one day prior to inoculation with C2VTrp1 tumor cells. Tumors were harvested on day 18 and stained for PD-1 and PD-L1 expression. (A) Representative flow gating scheme for determining PD-L1 and PD-1 expression on tumor and CD8 T cells respectively. (B) Percentage of zsgreen+ tumor cells expressing PD-L1 and (C) PDL1 MFI of tumor cells was quantified. (D) Percentage of PD-1+ of CD8+ T cells along with (E) MFI of PD-1-expressing CD8+ T cells was quantified. Statistical comparison was conducted via One-way ANOVA with Dunnett's multiple comparison test.

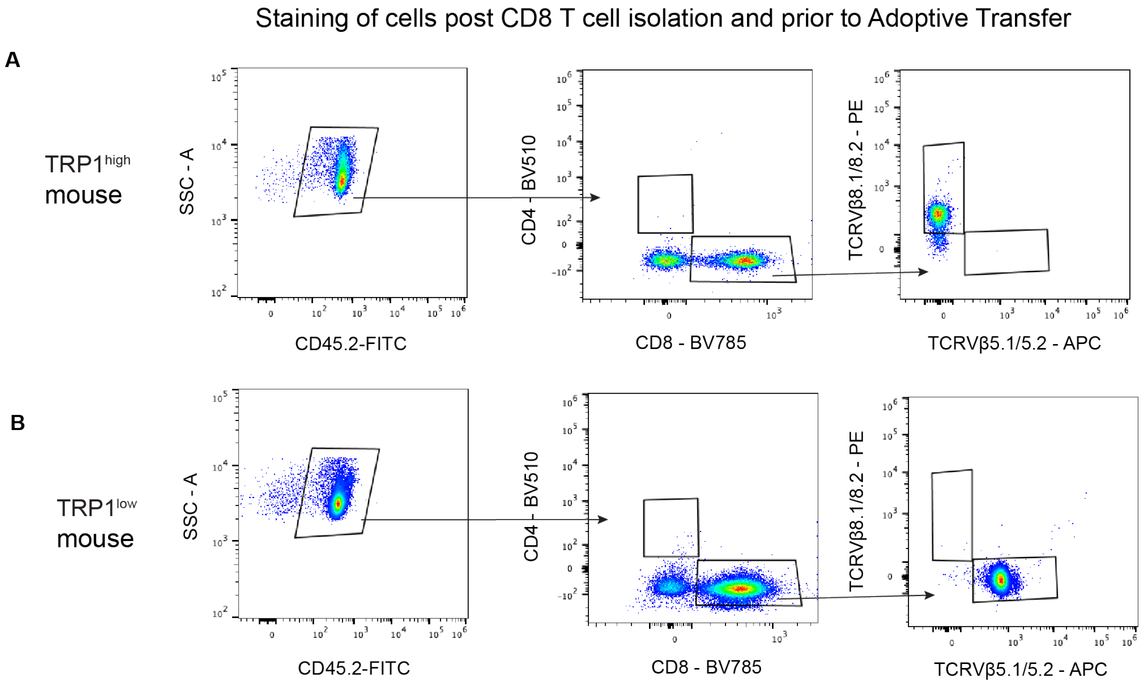

Fig. S9. CD8 T cells express TCRVβ8.1/8.2 from TRP1^{high} mice and TCRVβ5.1/5.2 from TRP1^{low} mice. (A) CD8 T cells were isolated from spleen and lymph nodes from TRP1^{high} or TRP1^{low} x Rag2-/- mice. TCR β variant chain expression was assessed for (A) TRP1^{high} and (B) TRP1^{low} prior to adoptive transfer into CD45.1+ recipient mice.

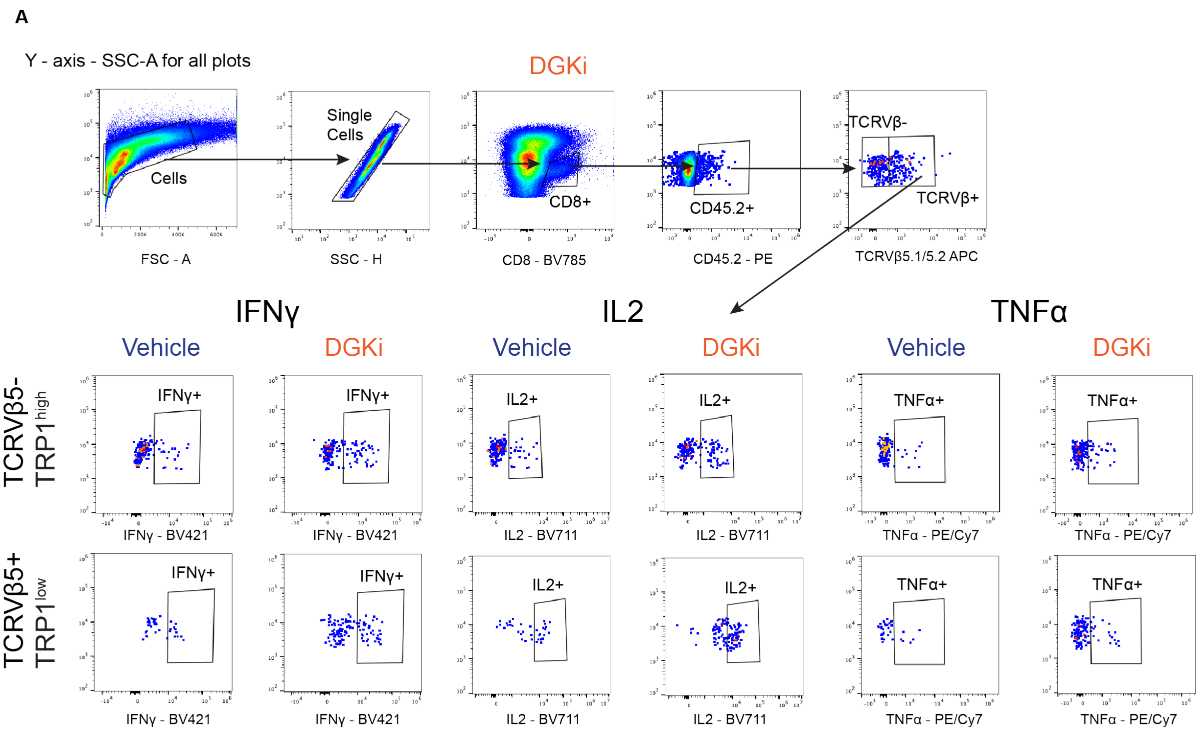

Fig. S10. Representative flow gating scheme for intracellular cytokine staining of C2VTrp1 tumors. (A) Single cells were gating on CD8 T cells followed by isolating the adoptively transferred TRP1^{high} and TRP1^{low} T cells through CD45.2+ staining. The two TRP1 T cells were differentiated by TCRVβ5 expression with TRP1^{low} positive for Vβ5 expression and negative for TRP1^{high} T cells. Subsequently, TRP1^{high} and TRP1^{low} cells were gated for their expression of IFNγ, IL2, and TNFα. Quantification of cytokine groups are shown in Fig. 7.

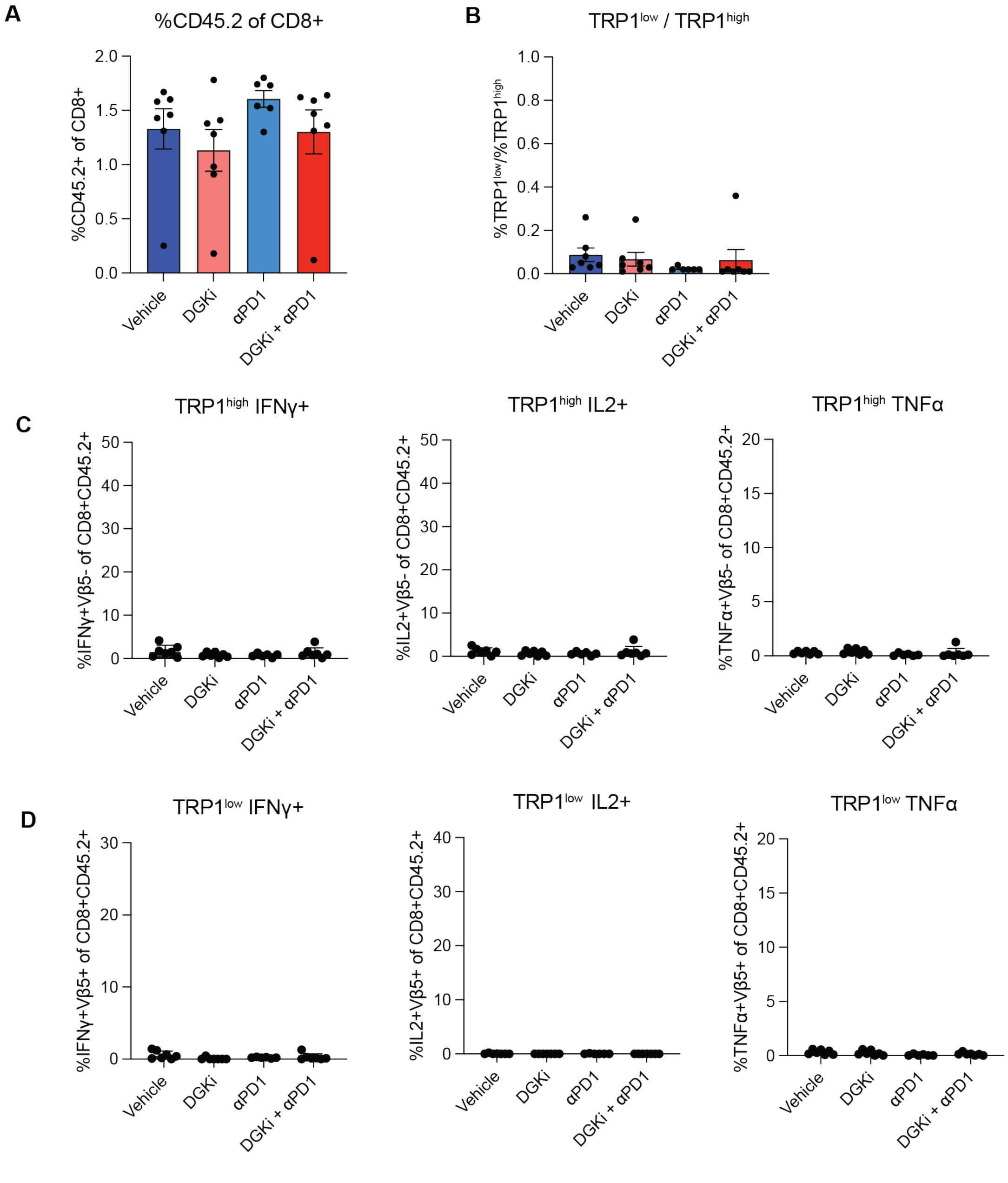

Fig. S11. Enhanced cytokine production by antigen-specific T cells in DGKi treated mice was not found in the spleen. (A) Percentage of adoptively transferred CD8 T cells found in the spleen from bearing C2VTrp1 tumors. (B) The ratio of $TRP1^{low}$ to $TRP1^{high}$ cells found in the spleen. Frequency of (C) $TRP1^{high}$ or (D) $TRP1^{low}$ producing IL2, IFNγ, and TNFα, was determined via intracellular cytokine staining. Errors bars represent SEM. Statistical comparison was conducted via one-way ANOVA and Dunnett's test for multiple comparison. No statistically significant comparisons were found.
